# Neurocognitive Assessment Tools for Military Personnel With Mild Traumatic Brain Injury: Scoping Literature Review

**DOI:** 10.2196/26360

**Published:** 2021-02-22

**Authors:** Chelsea Jones, Jessica Harasym, Antonio Miguel-Cruz, Shannon Chisholm, Lorraine Smith-MacDonald, Suzette Brémault-Phillips

**Affiliations:** 1 Heroes in Mind, Advocacy and Research Consortium Faculty of Rehabilitation Medicine University of Alberta Edmonton, AB Canada; 2 1 Field Ambulance Physical Rehabilitation Department Canadian Forces Health Services Department of National Defense Edmonton, AB Canada; 3 Faculty of Rehabilitation Medicine University of Alberta Edmonton, AB Canada; 4 Institute for Stuttering Treatment and Research Faculty of Rehabilitation University of Alberta Edmonton, AB Canada; 5 Department of Occupational Therapy Faculty of Rehabilitation Medicine University of Alberta Edmonton, AB Canada; 6 Glenrose Rehabilitation Hospital Research Innovation and Technology Glenrose Rehabilitation Hospital Edmonton, AB Canada

**Keywords:** military, rehabilitation, head injury, posttraumatic stress disorder, cognition, neurocognitive assessment tool, traumatic brain injury, assessment, brain concussion, mobile phone

## Abstract

**Background:**

Mild traumatic brain injury (mTBI) occurs at a higher frequency among military personnel than among civilians. A common symptom of mTBIs is cognitive dysfunction. Health care professionals use neuropsychological assessments as part of a multidisciplinary and best practice approach for mTBI management. Such assessments support clinical diagnosis, symptom management, rehabilitation, and return-to-duty planning. Military health care organizations currently use computerized neurocognitive assessment tools (NCATs). NCATs and more traditional neuropsychological assessments present unique challenges in both clinical and military settings. Many research gaps remain regarding psychometric properties, usability, acceptance, feasibility, effectiveness, sensitivity, and utility of both types of assessments in military environments.

**Objective:**

The aims of this study were to explore evidence regarding the use of NCATs among military personnel who have sustained mTBIs; evaluate the psychometric properties of the most commonly tested NCATs for this population; and synthesize the data to explore the range and extent of NCATs among this population, clinical recommendations for use, and knowledge gaps requiring future research.

**Methods:**

Studies were identified using MEDLINE, Embase, American Psychological Association PsycINFO, CINAHL Plus with Full Text, Psych Article, Scopus, and Military & Government Collection. Data were analyzed using descriptive analysis, thematic analysis, and the Randolph Criteria. Narrative synthesis and the PRISMA-ScR (Preferred Reporting Items for Systematic Reviews and Meta-analyses extension for Scoping Reviews) guided the reporting of findings. The psychometric properties of NCATs were evaluated with specific criteria and summarized.

**Results:**

Of the 104 papers, 33 met the inclusion criteria for this scoping review. Thematic analysis and NCAT psychometrics were reported and summarized.

**Conclusions:**

When considering the psychometric properties of the most commonly used NCATs in military populations, these assessments have yet to demonstrate adequate validity, reliability, sensitivity, and clinical utility among military personnel with mTBIs. Additional research is needed to further validate NCATs within military populations, especially for those living outside of the United States and individuals experiencing other conditions known to adversely affect cognitive processing. Knowledge gaps remain, warranting further study of psychometric properties and the utility of baseline and normative testing for NCATs.

## Introduction

### Background

Mild traumatic brain injuries (mTBIs), also known as concussions, are generally defined as a temporary change in brain functioning caused by an insult to the head, with a period of posttraumatic amnesia lasting less than a day [1]. Symptoms of mTBIs may include cognitive dysfunction, which can compromise the overall functioning at home and work and during other activities [2]. Within military populations, the mechanism of injury (MOI) for mTBIs varies, with some occurring as a result of motor vehicle collisions, falls, sports, explosions, or other forces related to combat and military training. Among Canadian Armed Forces service members deployed in Afghanistan during Operation Enduring Freedom, 5.2% self-reported experiencing an mTBI and, of these, 21% noted postconcussion symptoms (PCSs), referring to symptoms lasting longer than 3 months after MOI [1,3]. In comparison, studies among the US military populations reported mTBI rates of 12% to 22.8% during Operation Enduring Freedom and Operation Iraqi Freedom, with PCS rates of 15.8% to 35% [4-6]. The UK Armed Forces reported a 4.4% mTBI prevalence among service members deployed into these global conflicts [7]. Although the reported rates of mTBIs vary between militaries, the evidence base consistently demonstrates higher mTBI and PCS rates in military personnel versus civilian populations. Incidences of PCS are prevalent at an elevated rate among military populations, with global estimates for civilians of approximately 15% and military estimates ranging from 15.8% to 35% [5,7,8]. A higher prevalence of mental health disorders, exposure to traumatic experiences, and previous mTBIs among military personnel have been identified as potential reasons that PCS is more common in military populations than in civilian populations [3,5]. It is also accepted that factors such as stigma and fear of career repercussions owing to injury also contribute to the underreporting of mTBIs, which contributes to underestimating the actual incidence of this injury among military personnel [3,9].

### Neuropsychological Assessments in Military Populations

Premature return to duty after sustaining mTBIs is inherently associated with heightened risk. This includes increased chances of sustaining a subsequent concussion before neurological recovery. This has the potential to amplify the risk for impaired performance, making mission failure more likely, and endangering the safety of self and others [10,11]. Neurocognitive assessments for those who have sustained mTBIs are needed to (1) provide information on function in a timely fashion, (2) assist with diagnoses of mTBIs and/or impaired cognitive functioning, and (3) provide health care professionals with the tools needed to understand and monitor phases of recovery after injury for better-informed clearance for a return to work, duty, and other activities [12]. Measurement of neurobehavioral and cognitive functioning after mTBIs, often referred to as neuropsychological testing, is considered a component of best practice mTBI management. Neuropsychological assessments provide valuable information that can have important implications for returning to these activities in acute and chronic mTBI scenarios [1,2,13].

Traditional neuropsychological assessments are generally composed of measures with large normative databases and demonstrate evidence of adequate psychometric properties [13]. These assessments are typically administered in one-on-one scenarios by a trained health care professional with paper, pencil, and stopwatch [13]. Neuropsychological assessments range in administration time from less than an hour to multiple sessions over days. These assessments are not meant to be executed on the sidelines in athletic scenarios and are not simply screens of symptoms or cognitive status. Rather, they are in-depth assessments that address behavior, emotional status, and cognitive domains as well as neuropsychological symptoms. Neuropsychological assessments may or may not provide diagnostic information on mental health conditions, mTBIs, or learning disabilities; however, their diagnostic properties are still widely debated within the research [12].

Although neuropsychological assessments have been used in psychology for over 100 years, there remain many questions, logistical issues, and psychometric challenges around their use, especially in the military context. Traditional neuropsychological testing can be time intensive for both the health care professional and the patient, expensive for patients, and less feasible to administer in combat settings [13]. These cognitive assessments may also be dated. Some assessments use decades-old normative data. Others ask the patient to complete tasks that are no longer relevant to the present day. Dated assessment tools can compromise the validity of the assessment and increase the chances of type 1 and type 2 errors [14]. There are limits to the variations in stimuli that can be presented with traditional assessments and scoring (ie, speed and accuracy) and many of these assessments lack ecological validity [9]. Traditional neuropsychological assessments tend to examine isolated components or domains of cognition and may not adequately predict the overall functioning that relates to return to duty after mTBIs [9,14]. Assessment results do not always assist clinicians with treatment planning because performance during assessment tasks may not accurately reflect the real-world performance [14].

### Computerized Neurocognitive Assessment Tools

In the last 20 years, alternatives to traditional neuropsychological assessments have emerged in the form of computerized neurocognitive assessment tools (NCATs) [13]. As the use of computers and handheld devices such as tablets and smartphones has become ubiquitous in society, neuropsychological assessment tasks on these devices may be closer to activities that are commonplace in real life. This may increase the acceptability and ecolgical validity of computerized assessments.

NCATs developed in recent years are promising for use within military populations, especially with younger demographics. Currently, NCATs are used by military health care providers to assess the effects of mTBIs in both deployed and nondeployed settings [15]. In the United States, military personnel are mandated to undergo assessment with an NCAT referred to as the Automated Neuropsychological Assessment Metrics 4 Traumatic Brain Injury-Military (ANAM4 TBI-MIL) to establish a baseline of cognitive functioning before deployment in a war zone [16].

NCATs may have multiple benefits such as faster administration time, automated scoring and statistical analysis, easier reporting, and ease of deidentification of patients for research purposes [13,17]. NCATs may also allow for cognitive assessment to be obtained in geographic areas where traditional neuropsychological and cognitive assessment resources are limited [13]. Furthermore, NCATs provide the benefit of delivering numerous combinations of stimuli systematically and the ability to precisely track speed and accuracy. This can help mitigate practice effects and possibly increase sensitivity to subtle changes in cognitive performance [9,13]. The standardized tablet or computer interface, standardized script, and reduced conversation between the assessor and the participant may also enhance the interrater and intrarater reliability of NCATs [17]. Bias or issues with reliability that may be related to assessor variability or differences in rapport between the assessor and the patient may be reduced by standardized assessment delivery. Despite the potential benefits of NCATs, many questions remain regarding their effectiveness in both civilian and military populations with mTBIs and other conditions that affect cognitive functioning.

Although NCATs are currently used in military health care practices, a better understanding (or more information) about their feasibility, effectiveness and psychometric properties is needed. Owing to the relatively recent digital evolution, NCATs generally have not undergone the same degree of rigorous evidence-based psychometric evaluation as in traditional neuropsychological testing. Consequently, validity, reliability, specificity, and overall effectiveness may not be as well established for NCATs [13]. NCATs and traditional assessments may be limited regarding their ability to demonstrate cognitive functioning changes when individuals are immersed in stressful situations such as military combat; issues related to ecological validity also exist [9].

Diagnosing mTBIs on an individual basis has, to date, not been possible using a single traditional or computerized assessment. This diagnostic challenge can be attributed, in part, to large variations in baseline neurophysiological function and the presence of transient interferences such as learning effects, fatigue, anxiety, and unrelated states of mental alertness or illnesses [12]. Furthermore, although NCATs are being used in clinical settings and their utility in mTBI management is currently the subject of study, there is a lack of published literature on the use of these assessments among patients with other conditions known to adversely affect cognitive functioning [15].

### Previous Literature Reviews of NCATs

There have been a number of literature reviews published in the past 20 years, focusing on the usage of NCATs to assess sport-related mTBIs [13,18-20]. In 2005, Randolph et al [18] established 5 criteria that must be satisfied with additional research to consider an NCAT for testing after mTBIs. The *Randolph Criteria* included the following: (1) test-retest reliability, (2) the sensitivity of tests in the clinical issue of interest, (3) the validity of the measure, (4) reliable change scores and scoring algorithms for classifying impairment, and (5) determining the clinical utility of the measure [18]. The NCAT literature reviews of sport-related mTBIs after Randolph et al [18] have used these criteria. However, the most recent conclusions suggest that additional research is needed to further validate NCATs within mTBI populations [13]. These past literature reviews were not specific to military personnel.

It is essential that military personnel be considered a unique subset of the adult mTBI population for many reasons. First, military personnel exhibit higher rates of conditions such as posttraumatic stress disorder (PTSD), depression, anxiety, sleep disorders, chronic pain, substance abuse disorders, and mTBIs, which can cause and adversely affect the severity, longevity, and dysfunctionality of symptoms including associated cognitive dysfunction [3-7]. Specifically, traumatic brain injuries and PTSD can arise from the same or separate traumatic incidents and often co-occur, which adds complexity to the diagnosis, treatment, rehabilitation, and return-to-work planning [3-7,21]. Additionally, the MOI of the mTBI experienced by military members can differ from the impact sequelae seen in sport-related mTBIs. Blast injuries, for example, are more unique to military populations, with a portion of the mTBI sustained by military members during Operation Enduring Freedom and Operation Iraqi Freedom being attributable to this MOI [22,23]. A blast mTBI is an injury to the brain leading to dysfunction resulting from an explosion or a blast [22,23]. No significant variations in mTBI-attributed cognitive symptoms caused by blast versus blunt force have been identified; however, research continues to investigate this [22].

There is a need for the improved detection of neurocognitive deficits in the military setting to assist with the diagnosis of mTBIs, rehabilitation planning, tracking recovery, and making return-to-duty decisions while maintaining the productivity and safety of the military population and the civilians they may interact with at home and on deployment. An up-to-date scoping literature review of the current evidence related to NCAT usage among military members who sustained mTBIs is warranted because of (1) the lack of specificity to military populations among previous literature reviews regarding NCATs and mTBIs, (2) the rapid development of NCATs, and (3) the frequency of clinical usage among military health care. This scoping review aims to fill this knowledge gap.

### Purpose and Research Questions

The purpose of this scoping review is to (1) explore the existing evidence regarding the use of NCATs among military personnel who have sustained mTBIs, (2) evaluate the psychometric properties of the most commonly tested NCATs for this population, and (3) synthesize the data to explore the range and extent of NCATs among this population, clinical recommendations for use, and knowledge gaps requiring future research. This scoping review aims to answer the following research questions: (1) To what extent and which NCATs are being used within the military mTBI context? (2) What evidence exists regarding the validity, reliability, feasibility, technology acceptance, usability, and security of NCATs in the military and mTBI context? (3) What are the themes, clinical recommendations, and considerations in the evidence-based literature regarding the use of NCATs for military personnel who have sustained mTBIs? (4) What are the knowledge gaps and future directions of research that need to be addressed regarding the usage of NCATs for military personnel who have sustained mTBIs?

## Methods

### Scoping Literature Reviews

A scoping review is a form of knowledge synthesis that addresses an exploratory research question aimed at mapping key concepts, types of evidence, and gaps in research related to a defined area or field by systematically searching, selecting, and synthesizing existing knowledge [24]. While systematic reviews are used when answering narrowly focused research questions, scoping reviews are used to answer broad research questions. A scoping literature review is often conducted before the research begins and sets the stage for this research by highlighting gaps in the literature and explaining the need for research to be conducted [25]. Similar to a systematic review, an *a priori* protocol must be developed for a scoping review [26]. Unlike a systematic or critical review, and owing to the more iterative nature of a scoping review, deviations from the predetermined protocol may be necessary [26]. This evidence-based scoping literature review design is ideal for addressing the research questions and assisting with an evolving implementation science strategy to improve the cognitive assessments used with military populations.

This study employed the following overarching steps: (1) formulation of the research questions based on Population, Intervention, Comparison, and Outcome guidelines; (2) identification of relevant studies; (3) selection of studies; (4) charting of data; and (5) collation, analysis, summarization, and reporting of results [27]. As required for scoping reviews, a minimum of 2 reviewers were involved in study selection and analysis [27]. The PRISMA-ScR (Preferred Reporting Items for Systematic Reviews and Meta-analyses extension for Scoping Reviews) reporting guidelines were followed [28].

### Identification of Relevant Studies

Relevant studies were systematically identified. A description of the information sources, search strategy, inclusion and exclusion criteria, and selection process is provided in the following sections.

#### Information Sources and Search Strategy

A search strategy was developed based on specific inclusion and exclusion criteria and included the following databases: MEDLINE (Ovid MEDLINE ALL), Embase (Ovid interface), the American Psychological Association (APA) PsycINFO (Ovid interface), CINAHL Plus with Full Text (EBSCOhost interface), Psych Article (EBSCOhost interface), Scopus, and Military & Government Collection (EBSCOhost interface). The search consisted of an extensive list of keywords and subject headings covering 3 concepts: (1) NCATs, (2) military personnel, and (3) mTBIs. The 3 concepts were then combined with the Boolean AND. Studies were limited to peer-reviewed and gray literature papers in English. The initial search for papers took place on April 15 and April 21, 2020, within the aforementioned databases. The full search strategy is available in Multimedia Appendix 1.

#### Inclusion and Exclusion Criteria

Papers selected for inclusion in this study focused on military personnel who had a primary diagnosis of mTBIs. Targeted papers specifically addressed the usability, feasibility, reliability, validity, sensitivity, and efficacy of one or more NCATs among military personnel who have sustained mTBIs. Studies were excluded if the NCAT was used to measure the outcome of an intervention such as cognitive rehabilitation therapy, hyperbaric oxygen, or psychotherapeutic interventions. If the published work included healthy participants or participants with comorbid conditions, such as other mental health disorders, disrupted sleep, chronic pain, or substance use disorder, it was included if the additional conditions were secondary to the mTBI diagnosis and not the primary focus of the specific research study. Cognitive assessment practices that incorporated virtual reality were permitted for inclusion.

The papers included in the data set were quantitative, qualitative, mixed methods, and meta-analyses, regardless of positive, negative, or neutral findings. Papers were excluded from the review if they did not meet the inclusion criteria. Studies that exclusively addressed civilians or veterans were also excluded.

#### Selection of Studies

The study selection phases followed a variation of the procedures used by Miguel Cruz et al [29]. First, a member of the research team exported all of the identified studies to the reference manager software ProQuest Refworks. After deduplication, the references were imported into the Covidence Systematic Review Software. Second, members of the research team were trained in applying the inclusion and exclusion criteria (calibration phase) before the title and abstract evaluation phase. Three independent researchers evaluated the titles and abstracts of the remaining studies and compared them with the inclusion and exclusion criteria. Next, the research team met to resolve any differences in decisions to include or exclude studies from the review. During the full-paper reading phase, at least two researchers reviewed the full text of the selected studies. Each of the researchers independently assessed the studies to determine their suitability for inclusion in the data extraction phase. An article’s inclusion or exclusion into the data set for analysis required consensus from the research group. The reference lists of the included full-text studies were also reviewed for articles that the search may have missed.

### Charting of Data

The research team extracted data from the final selected papers according to the following domains: population (medical condition, age, specific military conflict, condition, race or ethnicity, sample size [N], and mean age [SD] in years), study features, clinical assessment, assessment of technology usability, technology outcome measures, technology, duration, and data analysis strategies. The researchers met regularly and reconciled the differences through discussion. In case of any disagreement, one of the researchers acted as a third rater.

### Analysis, Summarization, and Reporting

All data were analyzed and validated by at least two team members involved in the analysis. The research team met regularly to discuss data extraction, analysis, and synthesis, which were iterative and, in some cases, concurrent. Any discrepancies in the analysis of quantitative or qualitative data were resolved through discussion. This nonlinear process served to improve the rigor and internal validity of the review.

A narrative synthesis was conducted to organize, describe, and interpret the results of the analysis [30]. A deductive analysis was guided by the research questions associated with the use of computerized cognitive assessments among military personnel who have sustained mTBIs [31]. Inductive analysis was conducted from the information in the articles, particularly the recommendations and directions for future research. Furthermore, each of the 3 most common NCATs and their psychometric properties were considered within the 5 criteria proposed by Randolph et al [18]: (1) test-retest reliability, (2) the sensitivity of the tests in the clinical issue of interest, (3) the validity of the measure, (4) reliable change scores and scoring algorithms for classifying impairments, and (5) determining the clinical utility of the measure [18].

## Results

### Search Results

**Figure 1:**
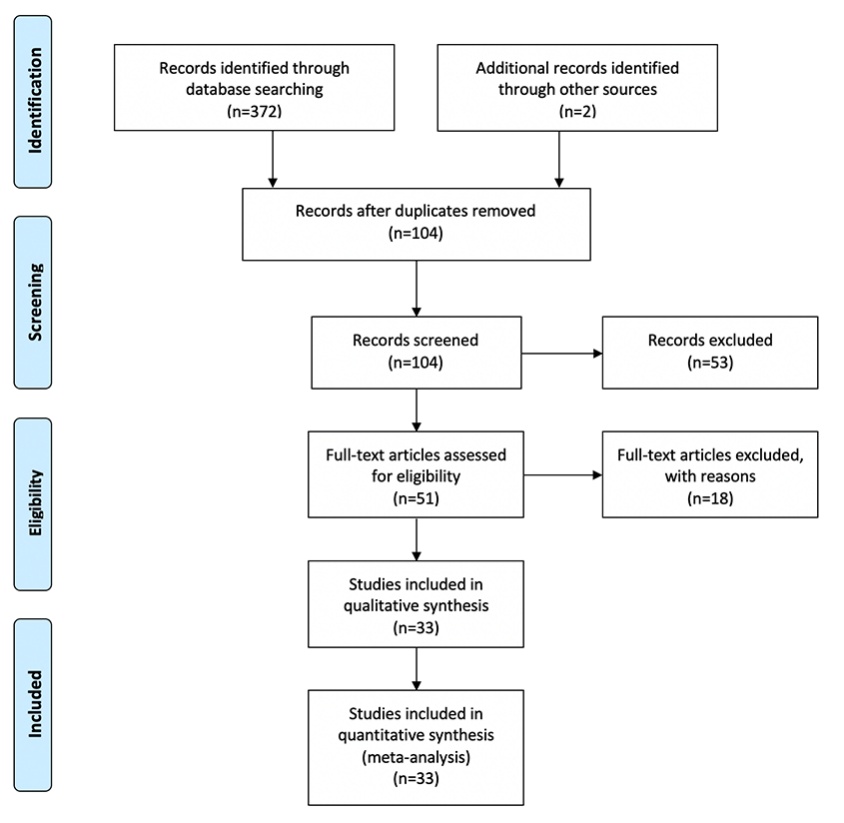
A PRISMA (Preferred Reporting Items for Systematic Reviews and Meta-Analyses) extension for Scoping Reviews chart of the scoping review study identification, selection, exclusion, and inclusion.

The search strategy yielded 372 articles (PRISMA diagram, Figure 1), with a further 2 studies identified through reference searches, resulting in a total of 374 articles. Following deduplication, 104 articles were subjected to a title and abstract review, after which 53 were removed. A total of 51 full-text documents were reviewed, with 18 being excluded for several reasons. Studies that were not specific to the military population, such as those focusing on veterans, pediatrics, caregivers, or athletes were excluded. Studies were also excluded if the research team was unable to verify that the neurocognitive assessment tool was computerized, the assessment tool exclusively evaluated reaction time, or if the primary condition evaluated was not an mTBI (eg, spinal cord injury, emotional distress, chronic traumatic encephalopathy, suicidality, or PTSD). The remaining 33 studies were included in the review. 

All included studies (n=33) were quantitative, and most studies were published in the United States and used the US military personnel as participants (Multimedia Appendix 2 [9,15,32-62]; Multimedia Appendix 3, Figures S2 and S3). The total number of participants included in the scoping review among all 33 studies was 36,872 (mean 1048.47, SD 2224.70), with an overall mean age of 27.31 (SD 4.10) years. Most participants were healthy (33,521/36,872, 90.90%) and male (31,587/36,872, 85.50%), with only 9.10% (3351/36,872) of all included participants having sustained an mTBI (Multimedia Appendix 3; Figure S6). While mTBI was the primary condition of interest, 3 studies included posttraumatic stress as a secondary condition of interest, and 2 studies included other nonspecified injuries (Multimedia Appendix 3; Figure S9). The time frame of the studies ranged from a single session to a 5-year follow-up.

A summary of the outcomes of the 33 included studies is presented in Multimedia Appendix 3. The outcomes vary greatly by research question, NCAT used, and study design.

The most commonly used NCATs among the 33 studies were the versions of the Automated Neuropsychological Assessment Metric (ANAM; 22/46, 46%), Defense Automated Neurobehavioral Assessment (DANA; 7/46, 15%), and Immediate Post-Concussion Assessment and Cognitive Testing (ImPACT; 5/46, 10%). A variety of secondary measures were collected across the studies, including other neuropsychological assessments and screens related to mTBIs (Multimedia Appendix 3; Figures S10 and S11). The quantitative study design used was not explicitly stated in all studies; however, most appeared to employ cross-sectional cohort designs (Multimedia Appendix 3; Figure S13). A multitude of constructs, including validity, temporal stability, and sensitivity, were measured using a variety of statistical methods throughout the studies, each with a unique purpose often specific to one NCAT (Multimedia Appendix 3; Figures S12, S14, and S15).

### Thematic Analysis and Narrative Synthesis

Thematic analysis and narrative synthesis revealed a number of topics related to the facilitators and barriers of NCAT usage among military populations. The 3 main themes that emerged through the studies included (1) comparing *apples to oranges*, (2) issues with validity, and (3) reliability issues. The narrative synthesis was framed in relation to the aforementioned criteria suggested by Randolph et al [18].

#### Comparing Apples to Oranges

A number of challenges around the approaches and comparisons used to establish the psychometric properties of the current NCATs were discussed across the included studies. Multiple studies noted that the comparisons made in research when assessing NCATs have important implications on the results and conclusions garnered from the current literature. These can have the potential to adversely affect reliability, validity, sensitivity, detection of reliable change, and overall clinical utility.

As a gold standard NCAT does not exist, comparisons between NCATs and traditional neuropsychological assessments are often used to determine how well tests relate to similar cognitive measures (convergent validity) and differ from dissimilar cognitive tests (discriminant validity) [32]. Simply adapting traditional neuropsychological tests to a computer platform fundamentally changes the test, rendering direct comparisons with the noncomputerized version inappropriate [32,63]. Some of the included studies aimed to address possible correlations with other traditional neuropsychological assessments and other secondary outcome measures related to a range of constructs (Multimedia Appendix 3; Figures S11 and S12).

Similarly, comparing different NCATs among each other can also be problematic when trying to establish validity. Although these assessments may aim to measure similar cognitive domains or constructs; they may measure or calculate scores for a particular construct differently [32]. One NCAT may measure a cognitive domain or construct with an individual subtest, whereas another NCAT may use an index score based on a combination of multiple subtests [32]. NCATs that use normative data, whether specific to military or general populations, have their own data set from which they generate standardized scores [32]. The variation among each NCAT makes it challenging for researchers to perform head-to-head comparisons or a hierarchy of these assessments.

The included studies compared the participants’ results with their baseline data, normative data, or both. When synthesizing findings across the studies included in this review, questions were raised regarding whether baseline or normative data comparisons were the most effective for establishing a change in performance among military personnel who had sustained mTBIs. Two papers specifically discussed this issue at length [15,33]. Baseline and normative data comparisons will be discussed further in the following theme and subsequent discussion.

Finally, even the comparison of those who have sustained mTBIs in healthy control groups can affect the results of studies related to NCATs. If some members of the mTBI group were asymptomatic, clinically meaningful differences between controls and those with symptoms could have been washed out, leading to limited effect sizes [32]. It was noted that very few studies in this study addressed within-group differences for the cohort with mTBIs.

#### Test-Retest Reliability

Four studies specifically addressed test-retest reliability for various NCATs among military populations. Dretsch et al [34] reported that, with the exception of the Simple Reaction Time (SRT) test, the ANAM had adequate or greater test-retest reliability values (intraclass correlation coefficient [ICC] 0.72-0.86) in the deployed environment, suggesting good temporal stability when the retesting interval is less than 11 days. Meyers [35] also addressed the temporal stability of the ANAM in a longitudinal study with follow-up sessions at 1, 3, and 5 years. In this study, the ICCs for all ANAM scales, except SRT1 and SRT2, showed ICCs of 0.7 to 0.8 [35]. Cole et al [36] found that of the ANAM, CogState, ImPACT, and CNS-Vital Sign (CNS-VS), each NCAT had at least one reliability score (ICC) in the *adequate* range (0.70-0.79) and only the ImPACT had a score that was considered high (0.80-0.89), with a time frame between tests of 30 days [36]. Using data from the previously mentioned study, Russo and Lathan [37] compared the DANA with the ANAM, CogState, ImPACT, and CNS-VS [37]. They found that the reliability coefficient measured for the DANA, when matching subjects across test and retest sessions, was higher than those for the ANAM and ImPACT [37]. These 4 studies conflicted in their conclusions on whether test-retest reliability was maintained over varying lengths of time.

Other types of reliability, such as internal consistency, were challenging to establish or judged to be not of adequate quality. Differences in the characteristics of test batteries, the design of test-retest studies, and insufficiently explained and nonstandardized methods of analysis makes it challenging to determine the reliability of NCATs [36,37]. Multiple studies reviewed indicated that the reliability coefficients of NCATs were below what would be considered clinically acceptable for clinical assessment [18,19,37].

#### Sensitivity of Tests in the Clinical Issue of Interest

One study specifically addressed the sensitivity of the DANA to detect small changes in neurofunctioning related to subconcussive blast pressure. LaValle et al [38] reported that the procedural reaction time (PRT) construct may have sufficient sensitivity to reliably detect a small, transient cognitive impairment among a healthy, undiagnosed population [38].

#### Validity of the Measure

Multiple studies included in the scoping literature review commented on issues with validity among NCATs both within military and nonmilitary populations who have sustained mTBIs. Studies discussed criterion, convergent, discriminant, and performance validity. Factors that can affect the construct validity of NCATs and traditional neuropsychological assessments include mental fatigue, physical environment, participant effort, practice effects, and the Monte Carlo effect, among others [34,39,40]. Test-retest reliability can affect validity and has been repeatedly found to be moderate at best and generally lower than that of NCATs [36]. Without an established gold standard, criterion validity cannot be established, which leads to the aforementioned issues caused by comparing NCATs with other NCATs or traditional neuropsychological assessments [32].

Some of the included papers discussed the threats to validity that arise with the variability of normative data sets in which NCAT scores are compared. For example, some NCATs, such as the ANAM, have normative data specific to the military population, whereas others, such as the ImPACT, are compiled from the general population. Some of the studies included in this scoping review discussed the problems that can occur when comparing military and civilian populations [15,33]. As previously discussed, military personnel may be more likely to experience mental health disorders, sleep disorders, chronic pain, substance abuse disorders, and other conditions that can adversely affect cognition and neuropsychological functioning than civilians. Observed decreases in neurocognitive performance following deployment in military personnel with and without mTBIs suggest that the environmental stressors of deployment may affect postdeployment neurocognitive performance [15,41]. Haran et al [15] noted that cognitive performance also correlates and changes with the deployment cycle as military members are more likely to have mental health challenges at specific times.

Recent evidence suggests that PCS is not specific to mTBIs and that symptoms following deployment are better accounted for by mental health diagnoses, such as PTSD, than by the history of mTBIs [3,7,9]. As it is common for healthy service members to have low scores on a cognitive test battery, it is beneficial to understand how many low scores and what cut-off scores are necessary to signify a clinically meaningful change for patients who have sustained mTBIs or other conditions [42]. Brenner et al [42] found that military members who screened negative for both posttraumatic stress and mTBIs had at least one low score on the ANAM shortly after returning from deployment. Another issue with normative data is the lack of consistency in demographic factors, as some are based on age and gender, whereas others are based on one or the other [40].

Many of the reviewed studies discussed the question of ecological validity among NCATs. Both NCATs and traditional neuropsychological assessments are typically administered in controlled clinical or research settings to obtain the best possible performance of the patient [9]. It is unknown if, and how well, this performance will transfer to a combat or deployed environment. It is also important to know how well executive functions measured during these assessments, such as decision making, translate to an individual’s performance in stressful situations. In the military context, where premature return to duty can have dire consequences, valid neuropsychological assessments that are specifically designed for use in military populations would be particularly useful [9]. The incorporation of virtual reality within NCATs may be a novel component to explore further [9]. NCATs with increased ecological validity would better assess operational performance and assist health care professionals in predicting risk for PCS and facilitating rehabilitation, recovery, and return-to-duty decision after mTBIs [44,45].

Four studies specifically addressed convergent, discriminant, and performance validity for various NCATs, including the Virtual Reality Stroop Test (VRST) [9], ANAM [39], CNS-VS, ImPACT, CogState [32], and single-item measures [46]. Armstrong et al [9] found that VRST significantly correlated with Stroop tests from other NCATs and traditional neuropsychological tests. It was also reported that the VRST conditions correlated significantly with the ANAM PRT and moderately with the ANAM Code Substitution [9]. Thomas et al [46] used signal detection item response theory models to provide initial validation of the Penn Face Memory, Test Penn Word Memory, and Test Visual Object Learning Test among US marines who had experienced mTBIs. Roebuck-Spencer et al [39] addressed an embedded performance validity measure for the ANAM that had moderate success [39]. This demonstrated the potential value of performance validity measures and sample-specific cut-off points in groups with cognitive impairments. Roebuck-Spencer et al [39] recommended higher cut-off points for those expected to have more severe cognitive impairments. Cole et al [32] found no clear patterns suggestive of convergent or discriminant validity between the 4 aforementioned NCATs.

#### Reliable Change Scores and Scoring Algorithms for Classifying Impairments

Only one study specifically addressed reliable change estimates for the ANAM4-TBI-MIL [47]. The authors suggested that reliable change cut-off scores and the base rates of meaningful change can be used to assist with the identification of postdeployment cognitive issues but should be interpreted with caution.

#### Determining the Clinical Utility of the Measure

Very few of the included studies addressed the clinical utility of the NCATs. Three studies addressed the usage of NCATs in varying environments, concluding that the DANA and the ANAM demonstrated comparable results and validity when comparing results from a controlled clinical setting with battlefield and deployment settings [41,48]. One study addressed the feasibility of using virtual reality for cognitive assessments [43]. The studies generally did not address issues of clinical utility such as acceptability, feasibility, security, appropriateness, practicability, accessibility, or usability from the perspectives of patients or health care professionals [64].

## Discussion

### Summarization of Findings

The purpose of this scoping literature review was to systematically explore the evidence regarding the use of NCATs among military personnel who have sustained mTBIs, evaluate the psychometric properties of the most commonly used NCATs for this population, and synthesize data around clinical recommendations for use, knowledge gaps, and future research directions. In total, 33 studies were included in this literature review, covering a range of constructs and topics related to NCATs. Three NCATs—the ANAM, ImPACT, and DANA—were the most commonly analyzed within the 33 studies.

This study was specific to military personnel who had sustained mTBIs. Many published articles have addressed the psychometric properties of NCATs, such as reliability, validity, sensitivity, and clinical utility, when used to assess cognition following mTBI in the general civilian population. Although this evidence was reviewed at length and used to lay the foundation of this paper, it did not meet the inclusion criteria to be included among the final 33 selected articles.

The 5 criteria proposed by Randolph et al [18] acted as a guide for evaluating the psychometric properties of the NCATs. Even with the paucity of information specific to military personnel with mTBIs, this criterion allowed consideration and discussion of the studies included in the scoping review and paves the way for making recommendations for future research on this topic.

For the first criterion of test-retest reliability, preliminary evidence demonstrates good test-retest reliability for the ImPACT and DANA among healthy military personnel and those with mTBIs and good-to-excellent test-retest reliability for the ANAM [34-37]. This finding is consistent with the reliabilities reported in the literature regarding mTBIs and sport-related mTBIs and are lower than desired for clinical decision making [36]. Although studies varied in test methods and time between testing, ICCs were promising for most of the constructs within these tests [34-37]. It must be noted that the time between testing sessions varied between studies, especially among the ANAM, and the available literature contains conflicting evidence on the length of time that temporal stability is maintained [34,37,65]. Additional studies addressing test-retest reliability with a standardized amount of time between tests and studies with larger sample sizes (especially for the DANA, ImPACT, CNS-VS, and CogSport) would assist health care professionals with clinical decision making regarding their choice of NCAT. The standardized time frame determined for test-retest reliability should be comparable with the recommended, eventual, and/or realistic use of NCATs.

The second criterion was the sensitivity of the tests in the clinical issue of interest. This was addressed by one study in relation to the DANA, which showed favorable results among a group of healthy male military members (n=202) [38]. Studies addressing sensitivity with NCATs among general populations have demonstrated good sensitivity of the ANAM, suggesting that this assessment [or this NCAT] has the potential to be used as a diagnostic tool for acute mTBI [13]. Two domains of the ImPACT that accurately classified individuals as concussed or not concussed with a high sensitivity and specificity were memory and speed [66]. Despite this finding, there is no universal evidence that the ImPACT adequately differentiates between healthy controls and individuals who have recently sustained mTBIs [13]. Studies have yet to demonstrate that NCATs have sufficient sensitivity to be used to accurately diagnose mTBIs or other conditions that affect cognition [13].

Validity is the most important aspect of test construction and must be considered when evaluating the clinical utility of a clinical assessment [13]. Owing to its importance, establishing the validity of a measure is the third criterion considered by Randolph et al [18]. For the ANAM, studies among military and general populations generally demonstrate some construct validity demonstrating that this NCAT is testing the constructs it was designed to test, although a review by Arrieux et al [13] stated that this was “*questionable at best*.” A study by Alsalaheen et al [67], using data from the general population, concluded that there is strong evidence for convergent validity of the ImPACT but weak or inconclusive evidence for discriminant validity, criterion validity, or diagnostic accuracy and utility, that is, there is evidence that NCATs measure similar cognitive constructs as traditional neuropsychological tests. Some evidence suggests that specific components of each NCAT can distinguish between individuals with acute concussion and healthy individuals or between individuals with and without mTBI symptoms [13]. Overall, the literature in this field is yet to provide definitive evidence in support of the convergent, discriminant, criterion, or internal validity of any of the NCATs included in this study [13]. It was also noted that predictive validity of future symptoms has yet to be established for any of the NCATs. Predictive validity would be an asset for health care professionals assisting patients during mTBI recovery [13].

The fourth criterion includes establishing reliable change scores and scoring algorithms for classifying impairment. Of the 33 studies, 1 study addressed reliable change scores for the ANAM; however, a reliable change index was established in 2018 for the ANAM using norms from the general population [47,65]. Reliable change criteria are lacking for many NCATs and should be addressed in future research with military and civilian populations to enable health care professionals to recognize meaningful changes in performance.

The fifth criterion, clinical utility, reveals the most significant knowledge gaps pertinent to patients, health care professionals, and health care organizations. Although the psychometric properties of any clinical outcome measure or assessment are important to establish among the population and condition in question, the discussion of feasibility, accessibility, acceptability, usability, appropriateness, specificity, and other factors is also equally important [64]. The results of the studies included in the scoping review are generally psychometric and research focused. The vast majority (n=30) of the papers reviewed highlighted knowledge gaps and recommendations for future research. However, facilitators and barriers to the usage of NCATs and clinical recommendations are generally absent from the papers.

Several additional issues were observed regarding the collective studies included in this study. First, the classification or diagnosis of mTBIs varied across all studies. Some studies relied on self-report to categorize participants into either an mTBI group or a healthy group. This practice is problematic for the following reasons. It is known that mTBIs and other injuries are widely underreported among military personnel. Participants may underreport mTBIs, whether intentionally or because they lack the health literacy to determine whether they have experienced a possible mTBI. It is also possible that some participants did not remember the event or pushed through it in a combat situation without recognizing it as an mTBI. Some studies either classified mTBIs as sustaining loss of consciousness or used symptom reporting to determine the incidence of mTBIs. Other studies used outcome measures with a set threshold to determine if participants would be in the mTBI or healthy group. Many of these outcome measures, which largely depend on self-reporting of somatic symptoms, do not have clear cut-offs to suspect mTBIs and do not have diagnostic utility. However, further studies reviewed medical records and relied on the diagnosis of mTBIs issued by a health care professional. The variability in methods and inclusion criteria for the mTBI group could affect validity and potentially facilitate the inclusion of those with mTBIs in the healthy group, which increases the chances of type 2 error.

The second concern highlighted by this review is the variation in whether or how the included studies screened participants for secondary conditions, such as depression, PTSD, fatigue and pain. These secondary conditions are known to adversely affect cognitive performance and could act as confounding variables [68]. Numerous studies have demonstrated that the severity of PTSD is negatively correlated with performance on multiple neuropsychological test batteries, including the DANA and ANAM, among military and civilian populations [68,69]. Some studies included in the scoping review explicitly stated that if a participant was enrolled in a military organization, it was assumed that he or she was a healthy individual, which may be an inaccurate assumption [68]. In the study by Brenner et al [42], participants with mTBI symptoms were screened for other conditions and were found to be significantly more likely to have a mental health diagnosis than those without mTBI symptoms. Given the evidence that PCS is not specific to mTBIs and that symptoms following deployment are better accounted for by mental health diagnoses rather than by mTBIs, researchers must consider the impact of neurobehavioral disorders that likely affect military members at a rate greater than that of the general population [5,68,69]. The implications of the increased occurrence of conditions that adversely affect the cognitive function among military populations may also change how normative data are used and interpreted for NCATs in research and clinical practice.

The baseline-referenced comparison approach has minimal supportive evidence from clinical trials but is the standard approach used in sports mTBI management and is favored by the US military, particularly with the ANAM [15,16]. Baseline referencing is thought to improve the sensitivity and specificity of NCAT scores as it controls for some intraindividual factors [15]. This approach is resource intensive and has multiple administrative and logistical barriers for many health care, athletic, and military organizations [15]. Normative referenced approaches are less resource intensive and require the establishment of a criterion-referenced standard to compare test results. Some NCATs use normative data compiled from the general civilian population, such as the ImPACT, whereas others, such as the ANAM, have multiple sets of norms, one of which is specific to the US military population [50,70-72].

When comparing results from a military cohort on the ANAM with both normative and baseline data, no statistical differences between the baseline-referenced approach and the norm-referenced approach for determining decrements in ANAM performance following mTBIs were observed [15]. In another study, no significant differences were found between the 2 approaches with the ANAM; however, both approaches were noted to be highly inconsistent in identifying military members who were found to have decreased cognitive performance, providing both false positives and negatives [33]. These findings suggest that there is no clear advantage of using the baseline-referenced approach over the norm-referenced approach.

In their 2017 paper, Coffman et al [68] considered the task of establishing a normative data set for the DANA in the context of the active-duty military population, focusing on which population-specific features should be accounted for in the process of defining a normative data set. This data set would consider the effect of conditions that adversely affect the scores of cognitive performance on NCATs. Extending beyond the issue of what population should be used to define normative neuropsychological data among active-duty military personnel, this study also recognized the challenge of identifying the features of a population to measure and control for ensuring that a normative data set truly represents the performance of normally functioning individuals [68].

Apart from the aforementioned occurrences of certain comorbidities within the military population, normative data based on a general adult civilian population tend to include wider age ranges from 18 to 85 years. The military population is much younger, often within the age range 18-60 years. Within this study, the average age of the participants included was 27.31 (SD 4.10) years, much younger than the normative age included in the general population norms. Studies addressing norm-based and baseline comparisons within military populations demonstrated variable results and raised more questions on best practices for clinical interpretation of cognitive performance scores on NCATs. This requires future consideration and research with military populations.

### Recommendations

A number of key recommendations were isolated from studies that are relevant for health care professionals. Most prominently, NCATs should be used cautiously and only as one source of information from among many other types of clinical tools and observations. It is not advisable that NCATs be used as a definitive or standalone diagnostic tool [40]. Cole et al [32] recommended that health care professionals should use the test they feel best fits their needs and targeted population for screening and follow-up assessments. Studies also noted that health care professionals should expect a decline in cognitive performance as age increases on the ImPACT and ANAM [49-51]. In addition, participants’ level of education may affect cognitive performance scores [49-51]. As the evidence-based literature on NCATs evolves, health care professionals must remain aware of forthcoming recommendations. Health care organizations and researchers will play an important role in translating this information promptly and accurately to facilitate improvements in clinical practice.

### Future Research

The findings of this scoping literature review have led to the formulation of the following recommendations for future research. First, it is apparent that more research is needed to better establish the psychometric properties of NCATs among military and civilian populations from a global perspective. Studies conducted in countries or military organizations outside of the United States are needed to assess constructs related to clinical utility within their specific contexts and populations. Research on the usage of NCATs within different deployment environments would also be beneficial. Furthermore, longitudinal studies that address temporal stability or test-retest reliability over time with different NCATs would be an asset. Studies that address the psychometric properties and clinical utility of NCATs with other conditions known to adversely affect cognitive functioning among military populations, such as depression, PTSD, sleep deprivation, chronic pain, and others, would be particularly beneficial. This would allow clinicians to better assess cognitive performance allowing them to make more informed clinical decisions. These decisions have the potential to influence the function, productivity, and safety of military members, their units, and those they interact with through their high-stake occupations. This would also assist clinicians in designing rehabilitation plans that target specific domains of cognition, leveraging cognitive strengths, and targeting areas of reduced performance.

Studies with a larger number of military personnel with mTBIs, or other conditions that affect cognition, would be an asset, especially for clarifying recovery trajectories and possibly return-to-duty decisions [45]. Future studies would be improved by applying a consistent definition and diagnoses of mTBI and related secondary conditions. It will also be important for future studies, particularly those focused on a specific condition, to test and control for other injuries or illnesses to minimize confounding variables.

Further research is also needed to better determine if using NCATs for baseline testing is indicated or if normative-based comparisons are valid for use in a clinical setting. Furthermore, the field would benefit from the establishment of standardized NCAT norms for military populations that represent not only healthy individuals but also those with mTBIs and other conditions that affect cognition.

Finally, studies that further address clinical utility, including the feasibility, accessibility, acceptability, usability, appropriateness, specificity, and other pragmatic factors, are needed to contextualize the use of NCATs and assist health care professionals with clinical decision making around which NCAT to use in practice, what rehabilitation is indicated, and how NCATs may guide the return-to-duty decisions. Evidence-based literature and guidelines on best practices that discuss facilitators, barriers, and recommendations for NCATs and digital health technologies would support health care professionals working with military personnel experiencing cognitive dysfunction.

### Strengths and Limitations

There are a number of notable strengths of this scoping review. This study was conducted following a planned a priori procedure, with attention to ensuring quality control and minimizing bias. The detailed search strategy was extensive, including 7 databases. The inclusion and exclusion criteria were determined before study onset and adhered to throughout. Appropriate calibration and pilot testing, use of at least two independent reviewers for all stages of the process, and group discussion of conflicts improved the quality of this scoping review.

Several limitations of this scoping review also warrant discussion. First, although the review process was calculated and rigorous, it is possible that relevant studies related to military personnel with mTBIs and NCATs were overlooked. Second, it is noted that other studies specific to civilian populations exist that were not included in this scoping review, which may include important information. Third, with the rapid rate of research and publishing on this topic, it is plausible that additional research has been published before the release of this scoping review. Finally, the limits of aggregate data and specific nuanced details may have become generalized during the synthesis process.

### Conclusions

Cognitive functioning is imperative to the day-to-day activities of military personnel in their work, self-care, and leisure activities. Military members must be able to make decisions in precarious and ambiguous situations where risk to self and others is high and must possess an adequate level of cognitive functioning to communicate, use weapons and technological devices, and perform other military duties without error. Assessing cognitive functioning is part of a multidisciplinary best practice protocol for the management and treatment of mTBIs [1,2,13]. NCATs are one such tool that can be used to assist health care professionals with treatment plans and guide recommendations about an individual’s readiness to return to activity.

The results of this study indicated that the published literature regarding NCAT usage among military personnel who have sustained mTBIs is quite heterogeneous in study design, construct being measured, and outcome goals. On the basis of the 5 Randolph Criteria [18], the psychometric properties of the most commonly evaluated NCATs among this population have yet to demonstrate adequate validity, reliability, sensitivity, and clinical utility for military personnel with mTBI. In addition, NCATs do not have the established diagnostic utility to identify which military members have sustained mTBIs and which have not. Additional research is needed to further validate NCATs within military populations, especially those outside of the United States and those who experience other conditions known to adversely affect cognitive processing. Further study of psychometric properties, clinical utility, and the utility of baseline and normative testing for NCATs is needed to assist health care professionals in improving clinical decision making and services for military personnel experiencing cognitive dysfunction.

## Appendix

Multimedia Appendix 1 Detailed search strategy.

Multimedia Appendix 2 Summary of included studies.

Multimedia Appendix 3 Detailed descriptive analysis of studies included in the scoping review.

## Abbreviations

ANAM: Automated Neuropsychological Assessment Metric
CNS-VS: CNS-Vital Sign
CogState: Axon Sports’ CogState Sport
DANA: Defense Automated Neurobehavioral Assessment
ICC: intraclass correlation coefficient
ImPACT: Immediate Post-Concussion Assessment and Cognitive Testing
MOI: mechanism of injury
mTBI: mild traumatic brain injury
NCATs: Computerized Neurocognitive Assessment Tools
OEF: Operation Enduring Freedom
PCS: postconcussion symptoms
PICO: Population, Intervention, Comparison, and Outcome
PRISMA-ScR: Preferred Reporting Items for Systematic Reviews and Meta-analyses extension for Scoping Reviews
PRT: prolonged reaction time
PTSD: posttraumatic stress disorder
SRT: simple reaction time
VRST: Virtual Reality Stroop Test
