# Supplementary material for: Neurocognitive Assessment Tools for Military Personnel With Mild Traumatic Brain Injury: Scoping Literature Review
Source: JMIR Ment Health. 2021 Feb 22;8(2):e26360. doi: 10.2196/26360 (PMC7939942; doi:10.2196/26360)
Supplement: Multimedia Appendix 1 [file mental_v8i2e26360_app1.docx]

## Multimedia Appendix 1: Detailed Search Strategy

Databases

1. Medline Ovid
2. Embase Ovid
3. Psycinfo Ovid
4. CINAHL Ebsco
5. Psyc articles Ebsco
6. Scopus
7. Military and Government Collection Ebsco

Search Terms

*Military*

(Military OR armed-force* OR armed-service* OR servicewomen OR servicemen OR air-personnel OR defense-force* or defence-force* OR service-personnel OR army OR navy OR air-force OR marine* OR sailor* or soldier* or infantryman or Civil-defense or Troops or ranger* or "medic" or coast guard or submariner* or active duty or enlisted personnel or reserve personnel).mp.

Computerized Cognitive assessment

((computer* or online or internet or web-based or ipad or tablet or tablets or smartphone* or cellphone* or electronic) adj10 (cogniti* or neuropsych* or neurofunction* or neurobehavio*) adj4 (assess* or screen* or measur* or test*)).mp.

(impact and (cogniti* or neuro*)).mp.

(Automated Neuropsychological Assessment Measure or ANAM or BrainFX or Brain FX or "Immediate Post-Concussion Assessment and Cognitive Testing" or Defense Automated Neurobehavioral Assessment or DANA).mp.

Mild traumatic brain injury

1. brain injuries, diffuse/ or brain injuries, traumatic/ or brain concussion/ or brain contusion/ or chronic traumatic encephalopathy/

2. (concuss* or postconcuss* or commotio-cerebri or coup-contrecoup or cranio-cerebral-trauma or ((brain or cerebral) adj5 contusion) or closed-head-injur* or mTBI or TBI or brain damage or brain injur* or head injur*).mp.

Medline

Date searched: April 15, 2020

Results: 56

1. (Military or armed-force* or armed-service* or servicewomen or servicemen or air-personnel or defense-force* or defence-force* or service-personnel or army or navy or air-force or marine* or sailor* or soldier* or infantryman or Civil-defense or Troops or ranger* or "medic" or coast guard or submariner* or active duty or enlisted personnel or reserve personnel).mp.

2. ((computer* or online or internet or web-based or ipad or tablet or tablets or smartphone* or cellphone* or electronic) adj10 (cogniti* or neuropsych* or neurofunction* or neurobehavio*) adj4 (assess* or screen* or measur* or test*)).mp.

3. (Automated Neuropsychological Assessment Measure or ANAM or BrainFX or Brain FX or "Immediate Post-Concussion Assessment and Cognitive Testing" or Defense Automated Neurobehavioral Assessment or DANA).mp.

4. 2 or 4

5. brain injuries, diffuse/ or brain injuries, traumatic/ or brain concussion/ or brain contusion/ or chronic traumatic encephalopathy/

6. (concuss* or postconcuss* or commotio-cerebri or coup-contrecoup or cranio-cerebral-trauma or ((brain or cerebral) adj5 contusion) or closed-head-injur* or mTBI or TBI or brain damage or brain injur* or head injur*).mp.

7. 6 or 7

8. 1 and 5 and 8

Psycinfo

Date searched: April 15, 2020

Results: 56

1. (Military or armed-force* or armed-service* or servicewomen or servicemen or air-personnel or defense-force* or defence-force* or service-personnel or army or navy or air-force or marine* or sailor* or soldier* or infantryman or Civil-defense or Troops or ranger* or "medic" or coast guard or submariner* or active duty or enlisted personnel or reserve personnel).mp.

2. ((computer* or online or internet or web-based or ipad or tablet or tablets or smartphone* or cellphone* or electronic) adj10 (cogniti* or neuropsych* or neurofunction* or neurobehavio*) adj4 (assess* or screen* or measur* or test*)).mp.

3. (Automated Neuropsychological Assessment Measure or ANAM or BrainFX or Brain FX or "Immediate Post-Concussion Assessment and Cognitive Testing" or Defense Automated Neurobehavioral Assessment or DANA).mp.

4. (exp Cognitive Assessment/ or neuropsychological assessment/) and exp Computerized Assessment/

5. 2 or 3 or 4

6. exp brain injuries/

7. exp head injuries/

8. brain damage/

9. (concuss* or postconcuss* or commotio-cerebri or coup-contrecoup or cranio-cerebral-trauma or ((brain or cerebral) adj5 contusion) or closed-head-injur* or mTBI or TBI or brain damage or brain injur* or head injur*).mp.

10. 6 or 7 or 8 or 9

11. 1 and 5 and 10

CINAHL Plus with Full Text

Date searched: April 15, 2020

Results: 59

1. (Military or armed-force* or armed-service* or servicewomen or servicemen or air-personnel or defense-force* or defence-force* or service-personnel or army or navy or air-force or marine* or sailor* or soldier* or infantryman or Civil-defense or Troops or ranger* or "medic" or coast guard or submariner* or active duty or enlisted personnel or reserve personnel)

2. ((computer* or online or internet or web-based or ipad or tablet or tablets or smartphone* or cellphone* or electronic) adj10 (cogniti* or neuropsych* or neurofunction* or neurobehavio*) adj4 (assess* or screen* or measur* or test*)) ) OR ( (Automated Neuropsychological Assessment Measure or ANAM or BrainFX or Brain FX or "Immediate Post-Concussion Assessment and Cognitive Testing" or Defense Automated Neurobehavioral Assessment or DANA) )

3. (MH “Brain Damage, Chronic”) OR (MH “Brain Injuryies+”) OR (MH “Head Injuries”) ) OR( (concuss* or postconcuss* or commotio-cerebri or coup-contrecoup or cranio-cerebral-trauma or ((brain or cerebral) N5 contusion) or closed-head-injur* or mTBI or TBI or brain damage or brain injur* or head injur*) )

4. S1 AND S3 AND S3

Embase

Date searched: April 21, 2020

Results: 69

1. (Military or armed-force* or armed-service* or servicewomen or servicemen or air-personnel or defense-force* or defence-force* or service-personnel or army or navy or air-force or marine* or sailor* or soldier* or infantryman or Civil-defense or Troops or ranger* or "medic" or coast guard or submariner* or active duty or enlisted personnel or reserve personnel).mp.

2. ((computer* or online or internet or web-based or ipad or tablet or tablets or smartphone* or cellphone* or electronic) adj10 (cogniti* or neuropsych* or neurofunction* or neurobehavio*) adj4 (assess* or screen* or measur* or test*)).mp.

3. (Automated Neuropsychological Assessment Measure or ANAM or BrainFX or Brain FX or "Immediate Post-Concussion Assessment and Cognitive Testing" or Defense Automated Neurobehavioral Assessment or DANA).mp.

4. 2 or 3

5. Exp diffuse brain injury/ or traumatic brain injury/ or exp concussion/ or brain contusion/ or chronic traumatic encephalopathy/

6. (concuss* or postconcuss* or commotio-cerebri or coup-contrecoup or cranio-cerebral-trauma or ((brain or cerebral) adj5 contusion) or closed-head-injur* or mTBI or TBI or brain damage or brain injur* or head injur*).mp.

7. 5 or 6

8. 1 and 4 and 7

Scopus

Date searched: April 21, 2020

Results: 57

( TITLE-ABS-KEY ( military OR armed-force* OR armed-service* OR servicewomen OR servicemen OR air-personnel OR defense-force* OR defence-force* OR service-personnel OR army OR navy OR air-force OR marine* OR sailor* OR soldier* OR infantryman OR civil-defense OR troops OR ranger* OR "medic" OR coast-guard OR submariner* OR active-duty OR enlisted-personnel OR reserve-personnel ) ) AND ( TITLE-ABS-KEY ( ( computer* OR online OR internet OR web-based OR ipad OR tablet OR tablets OR smartphone* OR cellphone* OR electronic ) W/10 ( cogniti* OR neuropsych* OR neurofunction* OR neurobehavio* ) W/4 ( assess* OR screen* OR measur* OR test* ) ) OR TITLE-ABS-KEY ( automated-neuropsychological-assessment-measure OR anam OR brainfx OR brain-fx OR immediate-post-concussion-assessment-and-cognitive-testing OR defense-automated-neurobehavioral-assessment OR dana ) ) AND ( TITLE-ABS-KEY ( concuss* OR postconcuss* OR commotio-cerebri OR coup-contrecoup OR cranio-cerebral-trauma OR ( ( brain OR cerebral ) W/5 contusion ) OR closed-head-injur* OR mtbi OR tbi OR brain-damage OR brain-injur* OR head-injur* ) )

Military and Government Collection

Date searched: April 21, 2020

Results: 36

military OR armed-force* OR armed-service* OR servicewomen OR servicemen OR air-personnel OR defense-force* OR defence-force* OR service-personnel OR army OR navy OR air-force OR marine* OR sailor* OR soldier* OR infantryman OR civil-defense OR troops OR ranger* OR "medic" OR coast-guard OR submariner* OR active-duty OR enlisted-personnel OR reserve-personnel

AND

( ( computer* OR online OR internet OR web-based OR ipad OR tablet OR tablets OR smartphone* OR cellphone* OR electronic ) N10 ( cogniti* OR neuropsych* OR neurofunction* OR neurobehavio* ) N4 ( assess* OR screen* OR measur* OR test* ) ) OR automated-neuropsychological-assessment-measure OR anam OR brainfx OR brain-fx OR immediate-post-concussion-assessment-and-cognitive-testing OR defense-automated-neurobehavioral-assessment OR dana

AND

concuss* OR postconcuss* OR commotio-cerebri OR coup-contrecoup OR cranio-cerebral-trauma OR ( ( brain OR cerebral ) N5 contusion ) OR closed-head-injur* OR mtbi OR tbi OR brain-damage OR brain-injur* OR head-injur*

Psych article

Date searched: April 21, 2020

Results: 39

1. (Military or armed-force* or armed-service* or servicewomen or servicemen or air-personnel or defense-force* or defence-force* or service-personnel or army or navy or air-force or marine* or sailor* or soldier* or infantryman or Civil-defense or Troops or ranger* or "medic" or coast guard or submariner* or active duty or enlisted personnel or reserve personnel).mp.

2. ((computer* or online or internet or web-based or ipad or tablet or tablets or smartphone* or cellphone* or electronic) adj10 (cogniti* or neuropsych* or neurofunction* or neurobehavio*) adj4 (assess* or screen* or measur* or test*)).mp.

3. (Automated Neuropsychological Assessment Measure or ANAM or BrainFX or Brain FX or "Immediate Post-Concussion Assessment and Cognitive Testing" or Defense Automated Neurobehavioral Assessment or DANA).mp.

4. (concuss* or postconcuss* or commotio-cerebri or coup-contrecoup or cranio-cerebral-trauma or ((brain or cerebral) adj5 contusion) or closed-head-injur* or mTBI or TBI or brain damage or brain injur* or head injur*).mp.

5. 2 or 3

6. 1 and 4 and 5
