# Supplementary material for: Neurocognitive Assessment Tools for Military Personnel With Mild Traumatic Brain Injury: Scoping Literature Review
Source: JMIR Ment Health. 2021 Feb 22;8(2):e26360. doi: 10.2196/26360 (PMC7939942; doi:10.2196/26360)
Supplement: Multimedia Appendix 2 [file mental_v8i2e26360_app2.docx]

## Multimedia Appendix 2: Summary of Included Studies (n=33)

| **Study** | **Study Design** | **Country** | **Population** | **Sex (%)** | **Mean Age (years ((SD))** | **Race (%)** | **Condition** | **Primary Assessment Utilized** | **Outcome of interest (NCAT) and construct outcome** |
| --- | --- | --- | --- | --- | --- | --- | --- | --- | --- |
| Adam, Mac Donald, Rivet, Ritter, May, Barefield, et al., (2015) | Quantitative: prospective, observational, cohort | USA | Military Members | Male (87.8) Female (12.2) | NR | NR | bTBI, healthy | ANAM4-TBI- MIL | PCS and performance on measures of posttraumatic stress disorder, depression, and neurocognitive performance at initial presentation correlate with return-to-duty time. Significantly greater impairment was observed in participants with mTBI vs controls. The largest effect size in ANAM performance decline was in Simple Reaction Time (SRT; p < 0.001). Time to return to duty correlated with ANAM SRT decline (r = 0.49, p < 0.0001). |
| Armstrong, Reger, Edwards, Rizzo, Courtney, Parsons, 2013 | Quantitative: validation study | USA | Military Members | Male (93.9) Female (6.1) | 28.78 (2.23) | White/Caucasian (51.0)  Black/African American (28.6)  Pacific Islander (4.1)  Other (14.3)  Ethnicity (not Hispanic) (81.6) | Healthy | Virtual Reality Stroop Test (VRST) | Results supported convergent and discriminant validity of the VRST. VRST was moderately correlated with the D-KEFS Stroop test and highly with the ANAM Stroop test. The VRST conditions had significant correlations with the ANAM Procedural Reaction Time (PRT) and moderate correlations with the ANAM Code Substitution (CS). VRST conditions not correlated with ANAM Simple Reaction Time (SRT1), Math Processing (MATH), Tower Test or PASAT. |
| Baker, Moring, Hale, Mintz, Young-McCaughan, Bryant, Broshek, 2018 | Quantitative: Cross-sectional cohort design | USA | Military Members | Male (86.0) Female (2.0) Missing (12.0) | NR | White/Caucasian (21.0)  Black/African American (1.5)  Hispanic/Latino (3.4)  American Indian/Alaskan Native (0.5)  Asian (0.6)  Native Hawaiian/Pacific Islander (0.2)  Other (1.1)  Unknown (60.1)  Missing (11.6) | bTBI, PTSD | ANAM4 | ANAM4 significantly correlated with the PTSD Checklist Military version (PCL-M; p < 0.01) and Acute Stress Disorder Scale (ASDS; p < 0.05). Only the ANAM4 SRT1 (p < 0.01) and SRT2 (p < 0.05) subtest scores significantly correlated with the Combat Exposure Scale (CES). Strongest correlations among the neuropsychological measures were between the Cognitive Stability Index (CSI) factors and the ANAM4 subtests (0.51 to 0.58; p < 0.05). |
| Betthauser, Brenner, Cole, Scher, Schwab, Ivins, 2018 | Quantitative: prospective, observational | USA | Military Members | Male (100.0) Female (0.0) | 26 (NR) | White/Caucasian (70.1) | mTBI, healthy, PTSD | ANAM4-TBI- MIL | SMs with PTS and/or mTBI performed worse on ANAM4 relative to controls with those with both conditions performing worst. Mean scores of these groups were generally in the average performance range (≥25th percentile) and well above cutoffs that are often deemed clinically meaningful in neuropsychology. Nearly one-third of soldiers who screened negative for both PTS and mTBI had at least 1 score that was unusually low or worse (<10th percentile) and 11.4% had a score that was extremely low (≤2nd percentile). |
| Brenner, Terrio, Homaifar, Gutierrez, Staves, Harwood, Warden, 2010 | Quantitative: exploratory, | USA | Military Members | Male (97.8) Female (2.2) | 24 (5.6) | White/Caucasian (73.0) Other (27.0) | bTBI, PTSD | PASAT, ANAM4-TBI- MIL | No significant differences between SMs with and without PTSD and/or mTBI were identified. Findings suggested differences in mTBI symptom reporting based on military rank, with fewer higher enlisted individuals reporting sequelae. Significant differences in years of education were noted between those soldiers with mTBI and PTSD and those with mTBI and no PTSD. |
| Bryan & Hernandez, 2012 | Quantitative: Cross -sectional cohort design | USA | Military Members | Male (92.2) Female (7.8) | 27.74 (7.07) | White/Caucasian (72.4) Black/African American (13.8) Hispanic/Latino (10.3) Asian, (1.7) Unreported (1.7) | mTBI | ANAM4 | SMs with TBI demonstrate greater declines in speed (χ2s > 8.541, Ps < .036, s > 0.271) and throughput (χ2s > 11.513, Ps < .009, s > 0.316) compared to SMs without TBI. Differences in accuracy were not significant (2s < 2.286, Ps > .131, s < 0.175). A significant proportion of SMs with TBI showed greater declines in speed across all ANAM subtests and greater than minimal declines on throughput SRT, PRT, Code Substitution-Learning (CLS), and spatial memory scores, with no significant differences on Code Substitution-Delayed (CSD) or Mathematical Processing (MATH). ANAM might be reasonably sensitive to TBI regardless of length of time from injury. |
| Coldren, Russell, Parish, Dretsch, & Kelly, 2012 | Quantitative: Cross -sectional cohort design | USA | Military Members | Male (88.4) Female (11.6) | NR | White/Caucasian (65.2) Black/African American (9.7) Hispanic (9.7) Other (12.3) | mTBI, healthy | ANAM4-TBI- MIL | Significant differences in changes from baseline scores for almost all ANAM subtests when mTBI are compared to non-mTBI. At follow-up, none of the ANAM subtests showed statistically significant differences between groups. All components of the ANAM normalize following a concussive injury in the combat setting, within 5 to 10 days. Results demonstrate the ANAM's lack of utility as a diagnostic or screening test beyond the first 10 days following a single, uncomplicated mTBI. |
| Cole, Arrieux, Dennison, & Ivins, 2017 | Quantitative: Cross -sectional cohort design | USA | Military Members | Male (79.4) Female (20.6) | 34 (NR) | White/Caucasian (66.5) Black/African American: (18.0) Hispanic (10.7) Other: (4.8) | Healthy | ANAM4-TBI- MIL, CNS-VS, CogState, ImPACT | Statistically significant order effects for CogState and CNS-VS and marginal or absent order effects for ANAM4 and ImPACT with no clinically meaningful implications. No significant differences (p < .05) for any of the NCATs. CogState appeared to be most impacted by order of administration. |
| Cole, Arrieux, Ivins, Schwab, & Qashu, 2018 | Quantitative: head-to-head | USA | Military Members | Male (83.1) Female (16.9) | 34.4 (7.9) | White/Caucasian (65.3)  Black/African American (16.7)  Hispanic (13.2)  Other (5.4)  Unknown (0.2) | mTBI, healthy | ANAM4-TBI- MIL, CNS-VS, CogState, ImPACT | 37 (0.6%) of the 5,655 correlations calculated between NCATs and neuropsychological tests are large (i.e., r ≥ 0.50). The majority of correlations are small (i.e., 0.30 > r ≥ 0.10), with no clear patterns suggestive of convergent or discriminant validity between the NCATs and neuropsychological tests. |
| Cole, Gregory, Arrieux, & Haran, 2018 | Quantitative: Cross -sectional cohort design | USA | Military Members | Male (86.0) Female (14.0) | NR | NR | mTBI, healthy | ANAM4-TBI- MIL | Mean and SD for the control group was significantly lower than the ISD (intraindividual standard deviation) mean for the mTBI group. Significant group differences in variability for control (F(1,13,678) = 848.65; p < .0001) and mTBI (F(1,13,678) = 1,815.71; p < .0001) groups. Significant interaction of group and time (F(1,340)= 15.87; p = .001; η2p = .03). The main effects for group (F(1,340) = 23.75; p = .001; η2p = .07) and time (F(1,340) = 15.87; p = .001; η2p = .05) were also significant and exceeded the recommended minimum for a practical effect. Significant main effect for time that exceeded the recommended minimum for a practical effect for the mTBI group only (F(1,340) = 11.49; p = .001; η2p = 0.10). |
| Cole, Arrieux, Schwab, Ivins, Qashu, & Lewis | Quantitative: head-to-head | USA | Military Members | NR | NR | NR | Healthy | ANAM4-TBI- MIL, CNS-VS, CogState, ImPACT | Each NCAT had at least one reliability score (ICC) in the “adequate” range (.70–.79), only ImPACT had one score considered “high” (.80–.89). Overall test–retest reliabilities in four NCATs in a military sample are consistent with reliabilities reported in the literature and are lower than desired for clinical decision-making |
| Connor, Dain Allred, Cameron, Campbell, Lauro, Houston, et al., 2018 | Quantitative: normative | USA | Military Academy | Male (76.1) Female 23.9) | 19.4 (1.5) | White/Caucasian (75.0) | Healthy | ImPACT | Significant, but small, sex effects were observed on the ImPACT visual memory task where females performed worse than males (p < 0.0001, pη2 = 0.01). While statistically significant differences may be observed on baseline tests, the effect sizes for competition and contact levels are very small, indicating that differences are likely not clinically meaningful at baseline. |
| Dretsch, Parish, Kelly, Coldren, & Russell, 2015 | Quantitative: Cross -sectional cohort design | USA | Military Members | Male (84.0) Female (16.0) | 26.4 (6.2) | White/Caucasian (62.5) Black/African American (11.3) Hispanic (6.3) Other (20.0) | Healthy | ANAM4-TBI- MIL | All but SRT (ICC = .57) had adequate or greater test-retest reliability (TRR) values (ICC =.72–0.86). ANAM has good temporal stability when the retesting intertrial interval is less than 11 days while in a deployed environment. |
| Haran, Alphonso, Creason, Campbell, Johnson, Young, & Tsao, 2013 | Quantitative: longitudinal | USA | Military Members | Male (100.0) Female (0.0) | 22.5 (3.4) | NR | mTBI, healthy | ANAM4-TBI- MIL | Significant differences in the total number of post-concussive clinical symptoms reported on the ANAM4-TBI-MIL from baseline. Declines in cognitive performance from the pre-deployment assessment (i.e., baseline) to the first post-deployment assessment which, except for SRT2, resolved by the second post-deployment assessment. Results suggest that cognitive declines during the chronic post-injury phase for some SMs with self-reported mTBI persist for periods as long as eight weeks post-deployment. |
| Haran, Alphonso, Creason, Campbell, Johnson, Young, & Tsao, 2016 | Quantitative: retrospective analysis | USA | Military Members | Male (100.0) Female (0.0) | 22.5 (3.4) | NR | mTBI, healthy | ANAM4-TBI- MIL | Both the mTBI and no mTBI groups performed similarly at baseline (ie, between; mean effect g = 0.05), and both groups had statistically significant decreases in scoring at follow-up testing when compared with their own group baseline (no MTBI groups, respectively. When performance was compared at follow-up, the mTBI group had significantly lower scores than the no mTBI group (ie, between) for 5 of the 7 subtests; however, the mean effect (g = 0.27) for these significant differences was below the recommended minimum practical effect size (RMPE) for group differences, suggesting no meaningful differences between groups. |
| Haran, Dretsch, & Bleiberg, 2016 | Quantitative: Cross -sectional cohort design | USA | Navy Service Members | Male (100.0) Female (0.0) | 34 (7.52) | NR | Healthy | DANA Brief | There were no significant practice effects observed for any subtest in any of the environmental conditions. No significant main effect for environmental condition (Λ = 0.996, F(3,10) = 0.14, p = .998, n2p = 0:004), suggesting there were minimal differences in mean throughput scores across the varying simulated environments. There were no significant differences between the simulated environmental conditions suggesting that performance on the DANA Brief is not impacted by thermal stress. No significant differences in performance within each simulated environmental condition associated with repeated administrations. |
| Haran, Dretsch, Slaboda, Johnson, Adam, & Tsao, 2016 | Quantitative: observational, retrospective analysis | USA | Military Members | Male (100.0) Female (0.0) | 29.1 (5.0) | NR | mTBI, healthy | ANAM4-TBI- MIL | There were no statistical differences, (p > 0.05), between baseline-referenced approach over norm-referenced approach for determining decrements in ANAM performance following mTBI. When the area under the curve for the ROCs were averaged across sub-tests, there were no significant differences between either the norm- referenced (0.65) or baseline-referenced (0.66) approaches (p > 0.05). |
| Hettich, Whitfield, Kratz, & Frament, 2010 | Quantitative: case review | USA | Military Members | Male (100.0) Female (0.0) | NR | NR | mTBI | ImPACT | Intra-test indicators demonstrated valid baseline and post-injury ImPACT assessments. One soldier had a complete resolution of symptoms and his ImPACT results returned to baseline within a couple of days, while the other took almost a week to see a return to baseline across all cognitive domains. |
| Iverson, Ivins, Karr, Crane, Lange, Cole, & Silverberg, 2020 | Quantitative: Cross -sectional cohort design | USA | Military Members | Male (100.0) Female (0.0) | Healthy: 28.2 (6.8) mTBI:26.9 (6.5) | White/Caucasian (64.8)  Black/African American (16.4)  Hispanic (13.2)  Other (5.6) | mTBI, healthy | ANAM4-TBI- MIL | Deficit scores showed larger group differences than the overall test battery mean (OTBM), but similar area under the curve (AUC) values. The deficit scores were highly correlated. All composites differed significantly between participants with and without mTBI (p < .001), with deficit scores showing the largest effect sizes (d = 1.32–1.47). Correlations between the symptom total score and the composite scores were all statistically significant and medium in size in the mTBI group. The correlations between cognitive symptoms and the composite scores were all statistically significant and small to medium in size in the mTBI group. |
| Ivins, Arrieux, Schwab, Haran, & Cole, 2019 | Quantitative: noninferiority study | USA | Military Members | NR* | NR* | NR* | mTBI, healthy | ANAM4-TBI- MIL, CNS-VS, CogState, ImPACT | SMs who performed at the worst level on any given NCAT also had low scores on the other NCATs they completed but not necessarily at an equally low level. These four commercially available NCATs that are similar and used for assessing patients with mTBI are sensitive in varying degrees to the effects of mTBI. The association between mTBI and low score level 1 was statistically significant (p > 0.05) for ANAM, CogState, and CNS-VS. The association between mTBI and low score level 1 was not significant for ImPACT (95% CI = 0.91–3.96) however, would have been statistically significant with a larger sample size. |
| Kelly, Coldren, Parish, Dretsch, & Russell, 2012 | Quantitative: validation study | USA | Military Members | Male (100.0) Female (0.0) | NR | White/Caucasian (74.0), Black/African American (6.0), Hispanic (17.0), Other (3.0) | mTBI, healthy | ANAM4-TBI- MIL | SMs with mTBI exhibited poorer performance than controls on all ANAM subtests, with significant differences on SRT, PRT, CS, and matching to sample (p <.001). Discriminant ability scores on SRT and PRT subtests was 71%, which improved to 76% when pre-deployment baseline scores were available. An exploratory clinical decision tool incorporating ANAM scores and symptoms improved discriminant ability to 81%. Results provide initial validation of the ANAM for detecting acute effects of battlefield mTBI. |
| Lathan, Spira, Bleiberg, Vice, Tsao, 2013 | Quantitative, comparison | USA | Military Members | NR | NR | NR | Healthy | DANA Standard, Rapid, Brief | DANA was found to be a reliable instrument and correlated favorably (p < 0.001; 0.85) to other computer-based neurocognitive assessments with the exception of the CSD task. ICC ranged from 0.88 to 0.95 with the exception of CSD (0.54) indicated excellent TRR. |
| LaValle, Carr, Egnoto, Misistia, Salib, Ramos, & Kamimori, 2019 | Quantitative: Cross -sectional cohort design | USA | Military Members | Male (100.0) Female (0.0) | 30 (5.5) | NR | Healthy | DANA Rapid | The neurocognitive task appearing most sensitive to identifying performance change is the PRT which may involve a sufficient level of challenge to reliably detect a small, transient cognitive impairment among a healthy undiagnosed population. Statistically significant fixed effects in PRT for Time (Est. = 9.1, 95% CI [0.5, 17.8]), Peak Overpressure (Est. = 11.8, 95% CI [4.5, 19.2]), Service (Est. = 1.6, 95% CI [0.1, 3.2]), and Sleep (Est. = −7.5, 95% CI [-13.6, −1.3]). Greater peak overpressure exposure, less sleep, and more military service years were associated with less PRT performance improvement. |
| Meyers, 2019 | Quantitative: longitudinal, cross-sectional cohort design | USA | Military Members | Male (90.9) Female (9.1) | 28.45 (6.81) | White/Caucasian (70.0)  Black/African American (13.1)  Hispanic (11.1)  Native (1.1)  Asian (2.0)  Other/mixed (2.4) | Healthy | ANAM4 | Results for individuals who were tested 1 year apart showed and at 3 years showed an ICC of .6 for SRT1 and SRT2 and .7 and above for all other scales. When the 5 year between testings data was examined, the ICCs for all scales except the SRT1 and SRT2 (ICC = .596) showed ICCs of .7 and.8 respectively. The ICC scores indicate that the ANAM scales appear stable over longer periods of time up to 5 years. |
| O’Connor, Dain Allred, Cameron, Campbell, Lauro, Houston, et al., 2018 | Quantitative: normative | USA | Military Academy | Male (76.1) Female 23.9) | 19.4 (1.5) | White/Caucasian (75.0) | Healthy | ImPACT | Significant, but small, sex effects were observed on the ImPACT visual memory task where females performed worse than males (p < 0.0001, pη2 = 0.01). While statistically significant differences may be observed on baseline tests, the effect sizes for competition and contact levels are very small, indicating that differences are likely not clinically meaningful at baseline. |
| Robitaille, Jackson, Hébert, Mercier, Bouyer, Fecteau, McFadyen, et al., 2017 | Quantitative: proof of concept | Canada | Military Members | NR | Healthy: 30.3 (5.3) mTBI: 30.3 (8.6) | NR | mTBI | VRai | VR was tolerated by both groups. Walking fluidity was significantly different between groups for the 2 hostile block (p < .046) between groups with a condition by group interaction (p < .04). Fluidity was degraded for the control group within the more complex navigational dual tasking involving avatars, and appeared greatest in the dual tasking with the interacting avatar. This navigational behaviour was not seen in the mTBI group. Findings show proof of concept for using avatars to expose differences in executive functioning when applying context-specific protocols. |
| Roebuck-Spencer, Reeves, Bleiberg, Cernich, Schwab, Ivins, Warden, et al., 2008 | Quantitative: Cross -sectional cohort design | USA | Military Members | Male (95.5) Female (4.5) | 23.17 (4.68) | White/Caucasian (61.1)  Black/African American (17.4)  Hispanic (14.9)  Native (1.2)  Asian (1.6)  Pacific Islander (1.1)  Other (2.7) | Healthy | ANAM3 | Performance differences between men and women were minimal on most ANAM subtests, but there was a clear speed/accuracy trade-off, with men favoring speed and women favoring accuracy on the Continuous Performance Test (CPT) subtest. Reaction time increased with age on most subtests, with the exception of MATH. Higher education resulted in significant but minimal performance increases on CDS, Matching to Sample (MSP), and Memory Search (STN) subtests. In contrast, substantial performance differences were seen between education groups on the MATH subtest. These data reveal that it is important to consider demographic factors, particularly age, when using ANAM to draw conclusions about military samples. |
| Roebuck-Spencer, Vincent, Gilliland, Johnson, & Cooper, 2013 | Quantitative: validation study | USA | Military Members, Civilians | Male (93.3) Female (6.7) | 29.7 (8.6) | White/Caucasian (58.3) Other (35.0) Unknown (6.7) | BI | ANAM4 | ANAM scores differed between groups with simulators scoring the highest. ROC curve analysis indicated excellent discriminability of ANAM scores ≥5 to detect simulators versus controls (AUC =0.858; odds ratio for detecting suboptimal performance =15.6), but resulted in a 27% false-positive rate in the clinical sample. When specificity in the clinical sample was set at 90%, sensitivity decreased (68%), but was consistent with other embedded effort measures. Results support the ANAM as an embedded effort measure and demonstrate the value of sample- specific cut-points in groups with cognitive impairment. |
| Roebuck-Spencer, Vincent, Schlegel, & Gilliland, 2013 | Quantitative: Cross -sectional cohort design | USA | Military Members | Male (91.0) Female (9.0) | 26.5 (6.4) | NR | Healthy | ANAM4-TBI- MIL | Overall rates of atypical performance were comparable across these 2 methods. However, these methods were highly discordant in terms of which individuals were classified as atypical. When norm-referenced methods were used, 2.6% of individuals classified as normal actually demonstrated declines from baseline. Further, 65.7% of individuals classified as atypical using norm-referenced scores showed no change from baseline (i.e., potential false-positive findings). |
| Roebuck-Spencer, Vincent, Twillie, Logan, Lopez, Friedl, Gilliland, et al., 2012 | Quantitative: Cross -sectional cohort design | USA | Military Members | Male (91.7) Female (8.3) | 26.2 (6.4) | NR | mTBI, healthy | ANAM4-TBI- MIL | All groups performed similarly at pre-deployment. The group reporting TBI with active symptoms performed worst at post-deployment and included the highest percentage of individuals showing significant decline in cognitive performance (30.5%). Of those reporting a TBI injury during deployment, 70% demonstrated no significant change in cognitive performance compared with baseline. 4.3% of controls showed significant decline in ANAM performance, compared to 18.5% of all SMs reporting a deployment-related mTBI. Performance decline in the mTBI group was statistically significant (X2 =42.4, p < .0001). Control group showed significant improvement in ANAM performance from pre- to post-deployment (p<.0001). |
| Russo & Lathan, 2015 | Qualitative: longitudinal | USA | Military Academy | Male (100.0) Female (0.0) | NR | NR | Healthy | DANA Rapid | The reliability coefficient measured for DANA, for matching subjects across test and retest sessions, is found to be higher than those from the ANAM and ImPACT, and comparatively, the DANA exhibits a reliability coefficient within tighter upper and lower bounds than both ANAM and ImPACT. The test–retest reliability of the DANA was found to be consistent between test and retest sessions administered within approx.77 days. The MD (minimum difference) for DANA per subtest is approximately 17% of the mean throughput, suggesting that with a small homogenous population in this time period (about 77days), test-retest reliability consistent for DANA. |
| Thomas, Brown, Gur, Moore, Patt, Risbrough, & Baker, 2018 | Quantitative, longitudinal | USA | Military Members | Male (100.0) Female (0.0) | NR | White/Caucasian (91.0) Black/African American (4.0)  American Indian/Alaskan (2.0)  Asian (2.0)  Hawaiian/Pacific Islander (1.0) | TBI | PFMT, PWMT, VOLT | Signal detection–item response theory (SD-IRT) models adequately fitted recognition memory item data across all modalities. Face and word memory tests had two meaningful dimensions along which individual differences could be characterized. The object learning data appeared to have just one meaningful dimension. |
| Vincent, Bleiberg, Yan, Ivins, Reeves, Schwab, Warden, et al., 2008 | Quantitative: normative | USA | Military Members | Male (91.0) Female (9.0) | 26.0 (5.8) | NR | Healthy | ANAM3 | Variability of the performance measures between genders differed according to age for all tests (p < 0.01). A general decline in performance with age should be expected on most tests in the ANAM battery. |
| Wright, Handy, Avcu, Ortiz, Haran, Doria, & Servatius, 2018 | Quantitative: Cross -sectional cohort design | USA | Navy Service Members | Male (80.6) Female (19.4) | Healthy: 25.95 (4.48) mTBI: 33.57 (7.93) | NR | Healthy | DANA Standard | No significant difference in overall neurocognitive performance as a function of lifetime mTBI (F(9, 21) = 1.52, p = 0.21). No group differences in throughput for any single neurocognitive assessment contained in the DANA battery (p < 0.05). No group difference in overall neurocognitive performance for any single neurocognitive task (all p < 0.05). |
