# Supplementary material for: Neurocognitive Assessment Tools for Military Personnel With Mild Traumatic Brain Injury: Scoping Literature Review
Source: JMIR Ment Health. 2021 Feb 22;8(2):e26360. doi: 10.2196/26360 (PMC7939942; doi:10.2196/26360)
Supplement: Multimedia Appendix 3 [file mental_v8i2e26360_app3.docx]

## Multimedia Appendix 3: Detailed Descriptive Analysis of Studies Included in Scoping Review

*Figure S1: Publication years of papers included in this scoping review (n=33)*


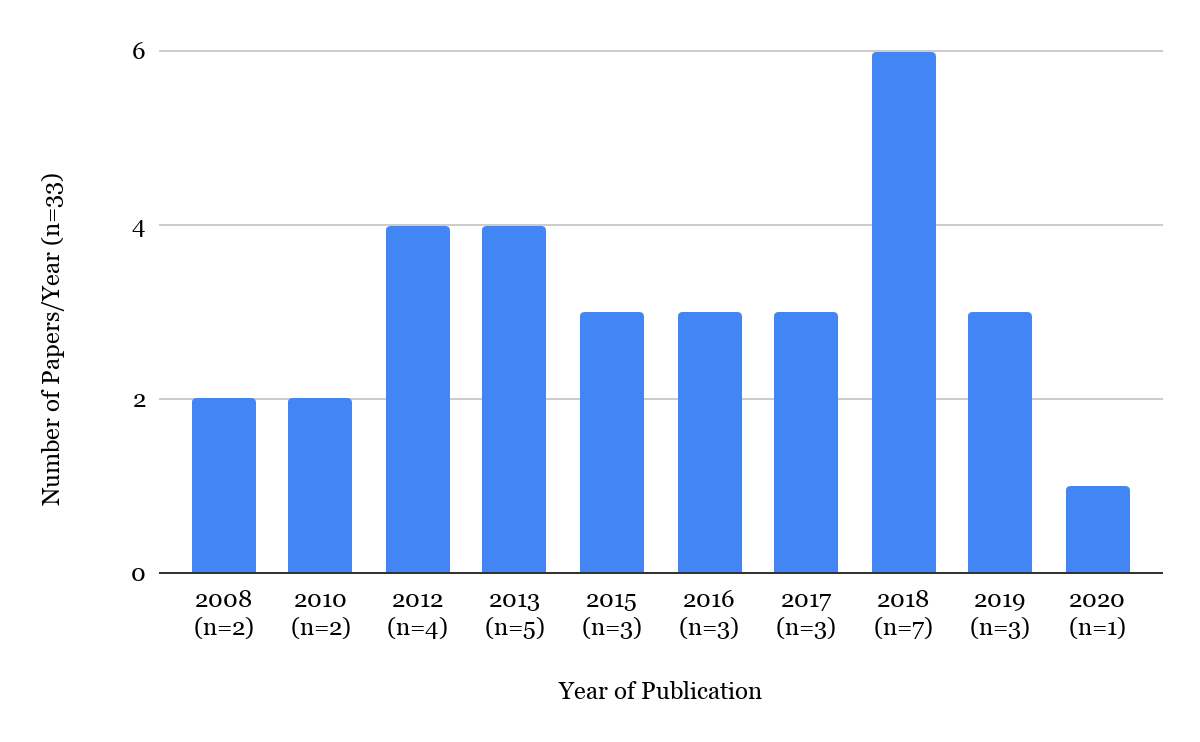


*Figure S2: Country of origin of included military participants (n=34)*


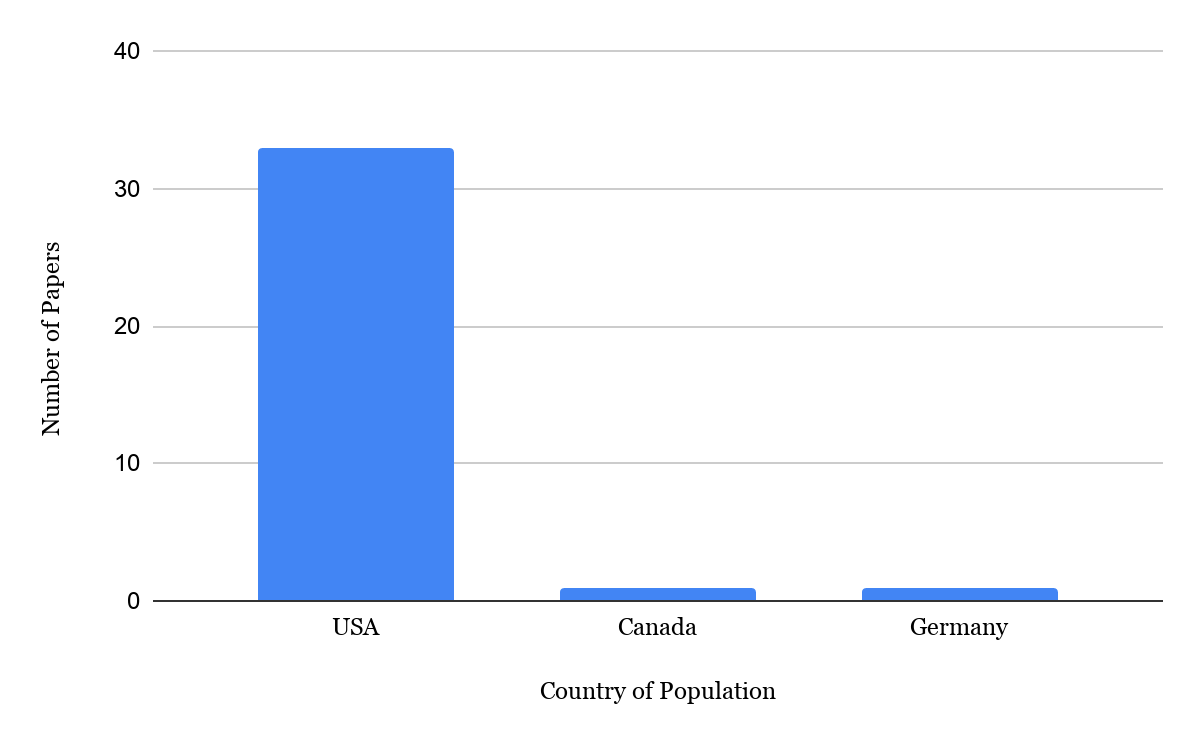


*Figure S3: Country of paper publication (n=33)*


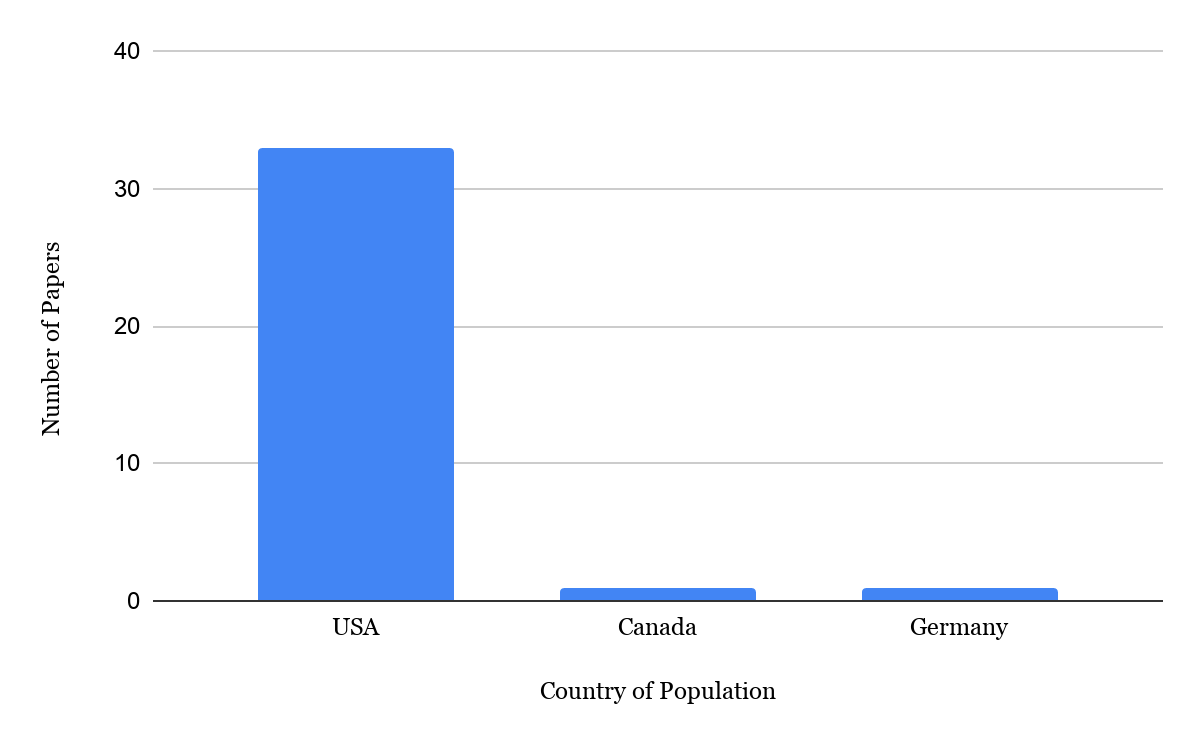


*Figure S4: Categories of participants included in scoping review by paper (n=34)*


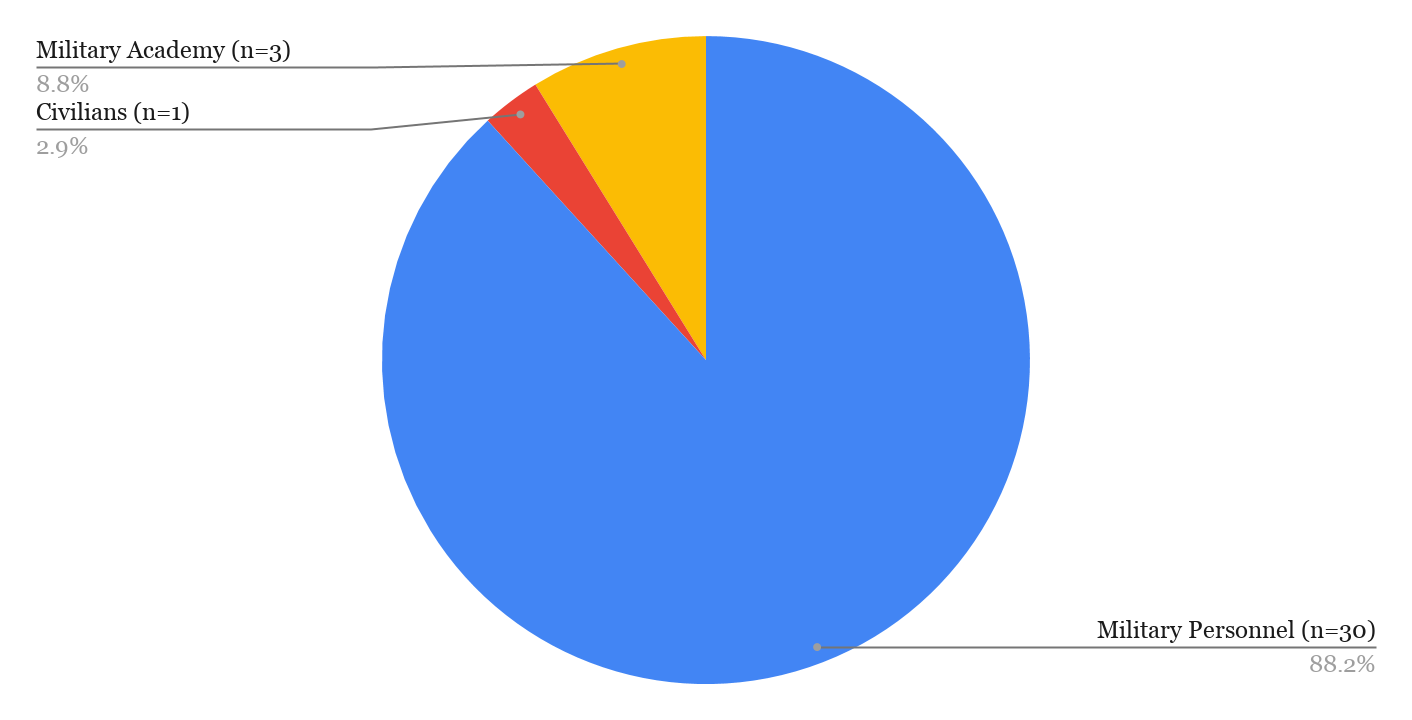


*Figure S5: Sex of the participants included in scoping review (n=36,657)*


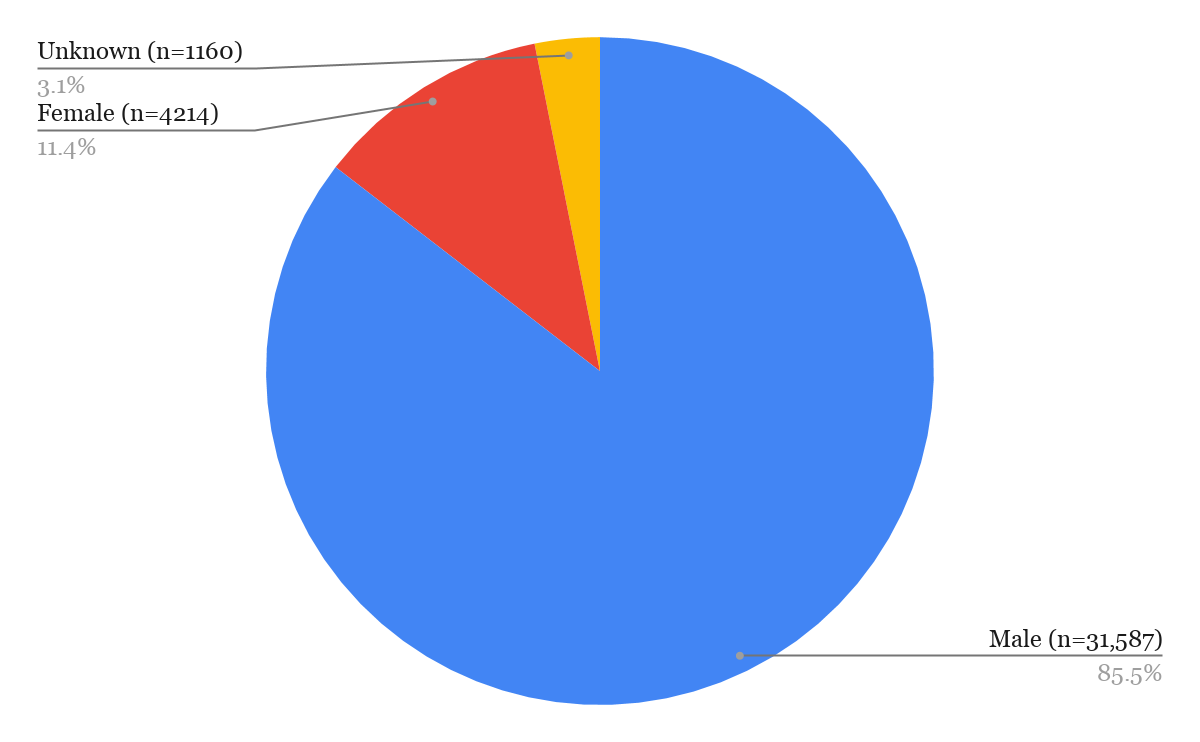


*Figure S6: Specific conflicts of papers included in scoping review (n=32)*


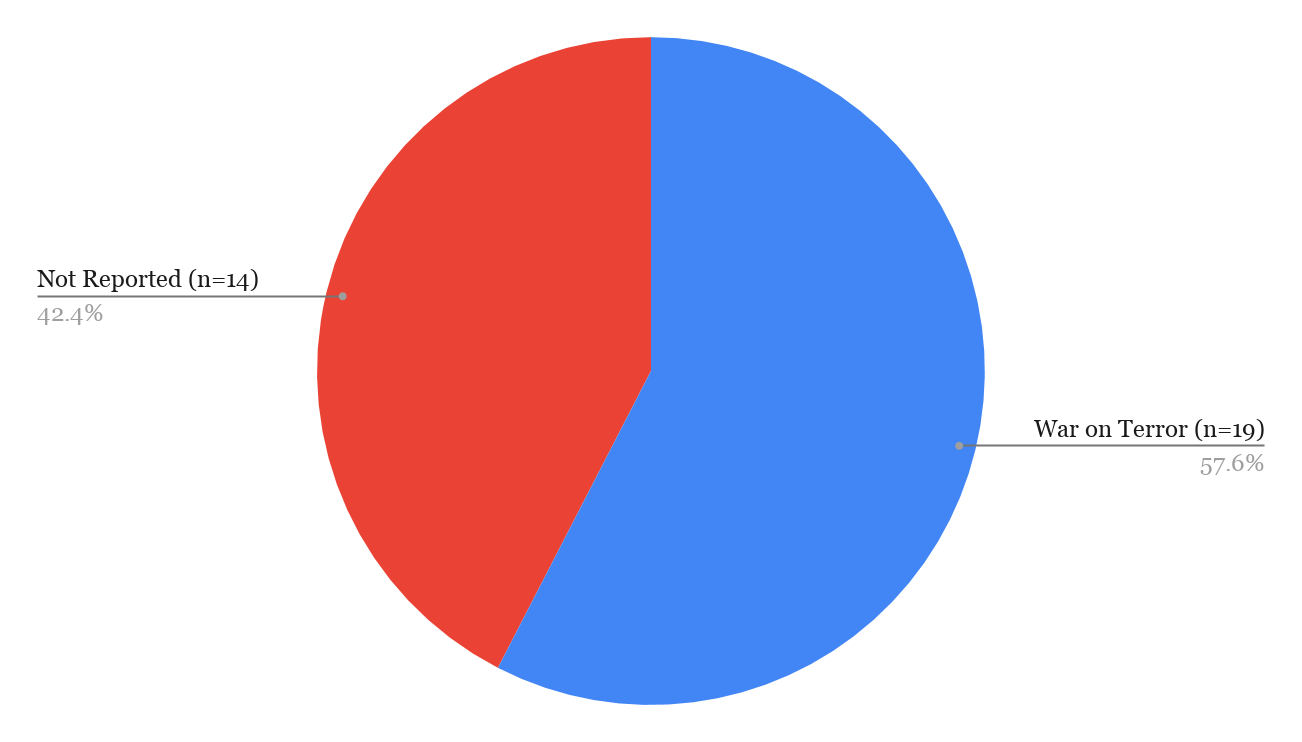


*Figure S7: Primary condition of participants included in scoping review (n=36,872)*


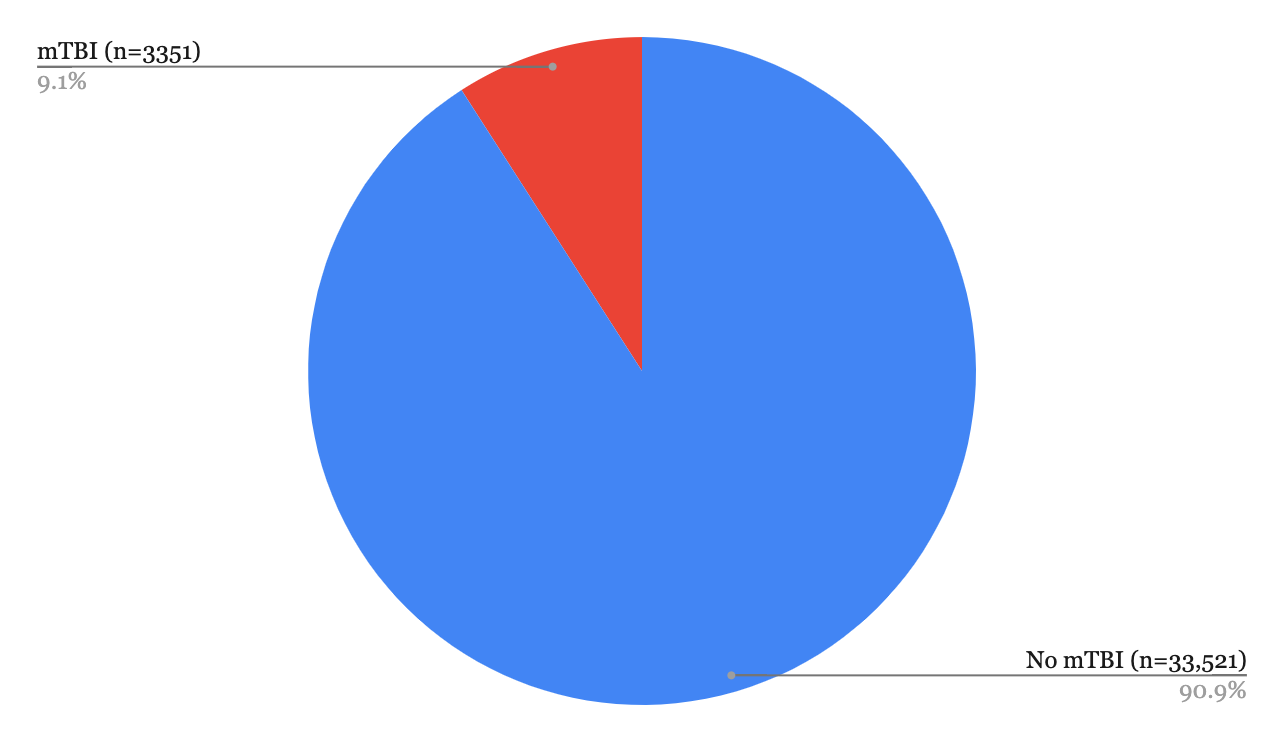


*Figure S8: Primary Impairment Category ICD-10 (n=44)*


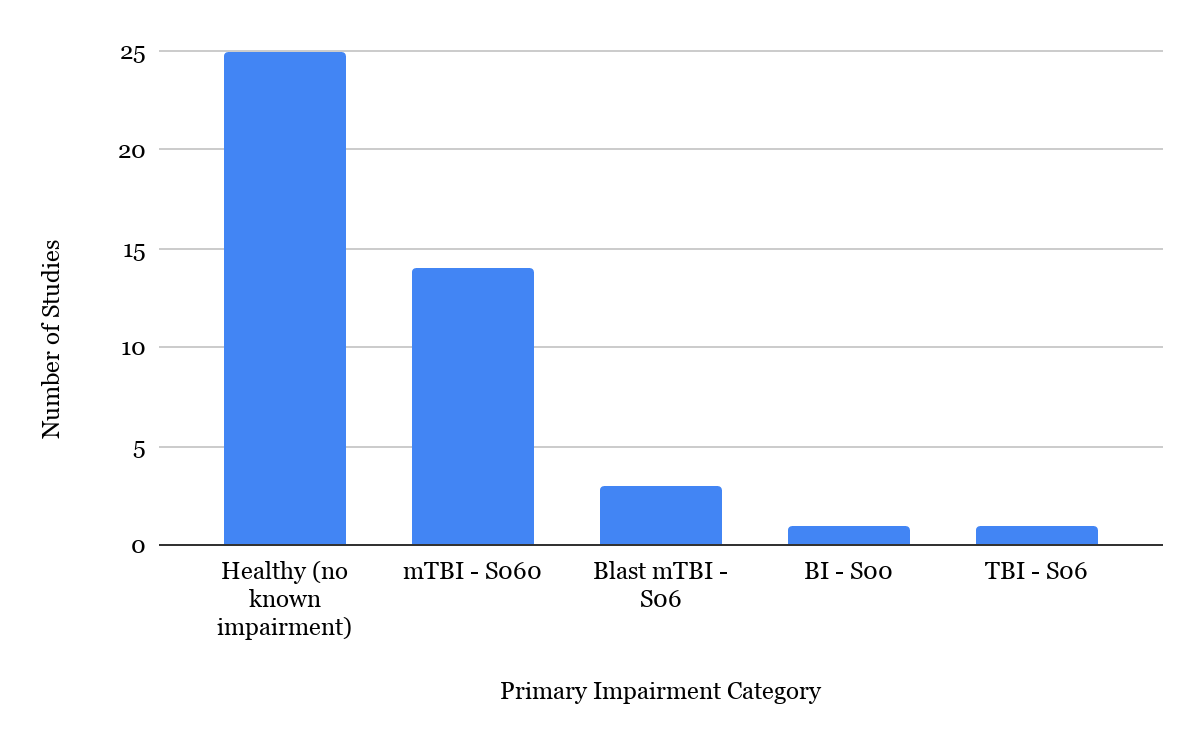


*Figure S9: Secondary Impairment Category ICD-10 (n=33)*


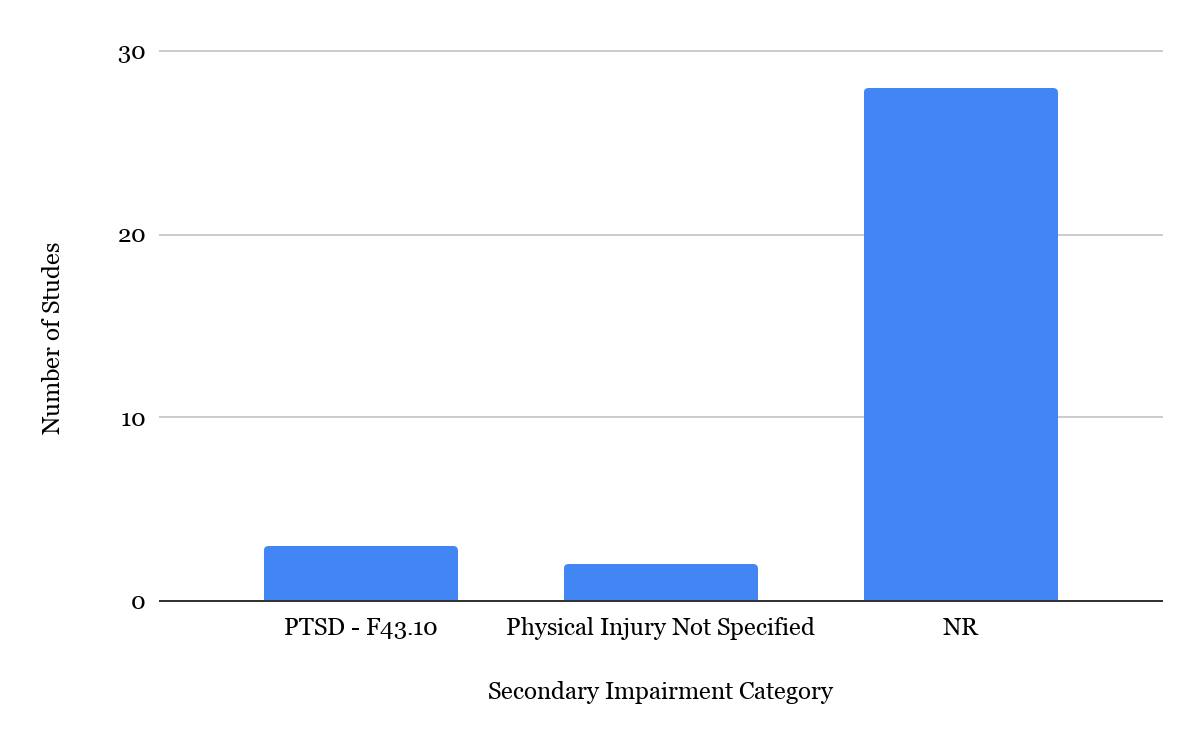


*Figure S10: NCAT type by paper (n=46)*


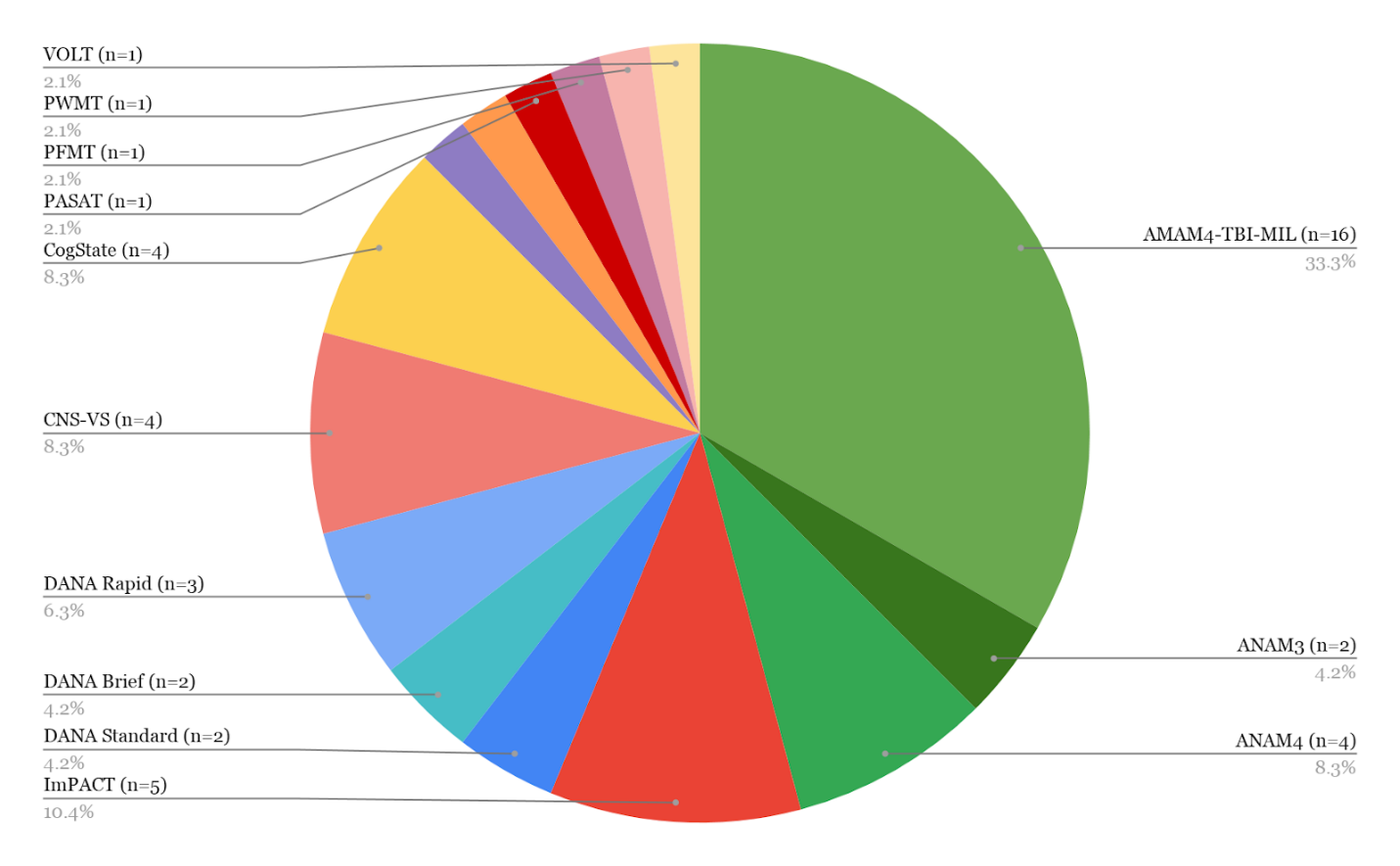


ANAM4-TBI- MIL (Automated Neuropsychological Assessment Metrics Version 4 Traumatic Brain Injury Military); ANAM3 (Automated Neuropsychological Assessment Metrics Version 3); ANAM4 (Automated Neuropsychological Assessment Metrics Version 4); ImPACT (Immediate Post-Concussion Assessment and Cognitive Testing); DANA Standard (Defense Automated Neurobehavioral Assessment Standard); DANA Brief (Defense Automated Neurobehavioral Assessment Brief); DANA Rapid (Defense Automated Neurobehavioral Assessment Rapid); CNS-VS (CNS Vital Signs); CogState (Axon Sports’ CogState Sport); VRST (Virtual Reality Stroop Test); VRai (Virtual Reality avatar interaction); PASAT (Paced Auditory Serial Addition Test); PFMT (Penn Face Memory Test); PWMT (Penn Word Memory Test); VOLT (Visual Object Learning Test).

*Figure S11: Secondary outcome measures (n=46)*


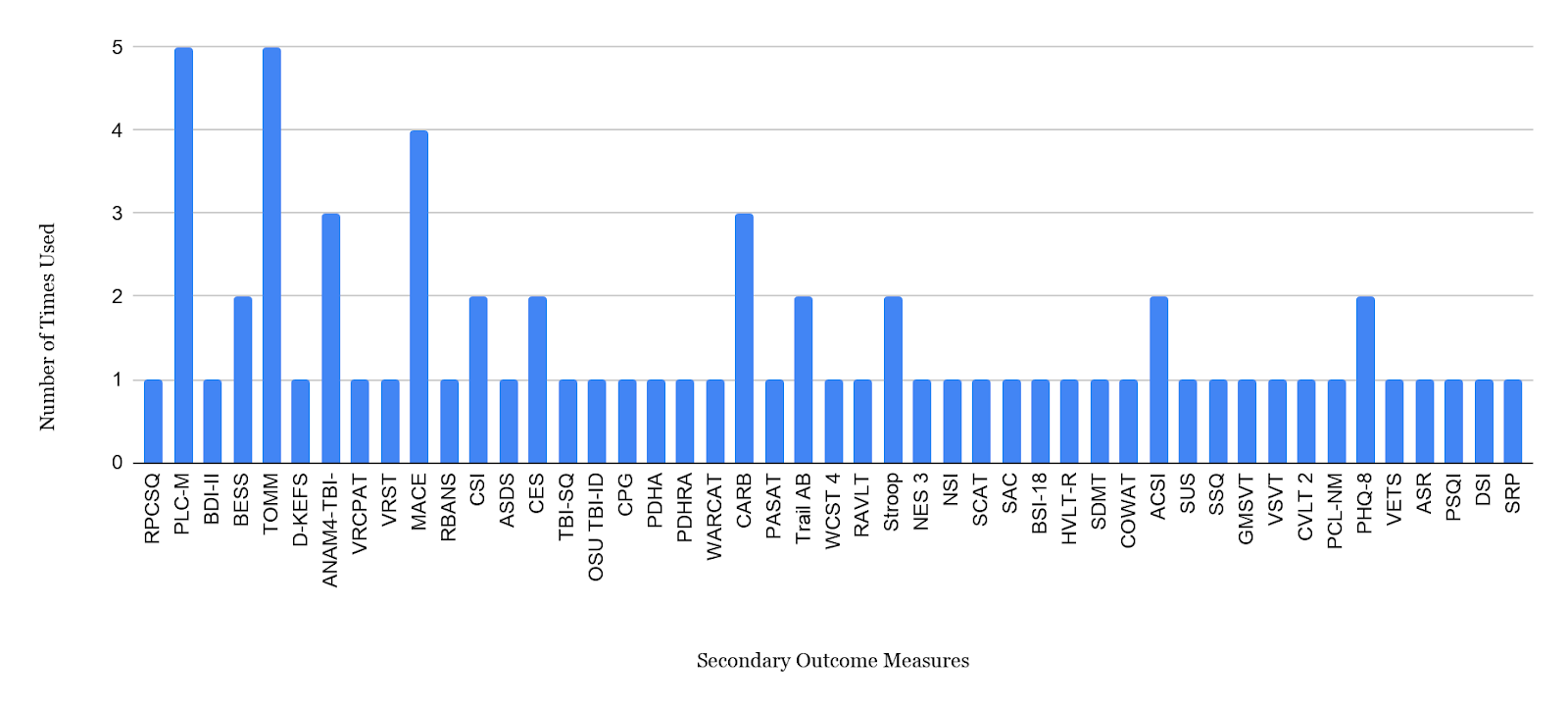


RPCSQ (Rivermead Post Concussion Symptom Questionnaire); PCL-M (Posttraumatic Stress Disorder Check List-Military); BDI-II (Beck’s Depression Inventory II); TOMM (Test of Memory Malingering); BESS (Balance Error Scoring System); D-KEFS (Delis-Kaplan Executive Function System); ANAM4-TBI- MIL (Automated Neuropsychological Assessment Metrics Version 4 Traumatic Brain Injury Military); VRCPAT (Virtual Reality Cognitive Performance Assessment Test); MACE (Military Acute Concussion Evaluation); RBANS (Repeatable Battery for the Assessment of Neuropsychological Status); CSI (Cognitive Stability Index); ASDS (Acute Stress Disorder Scale); CES (Combat Experience Scale); TBI-SQ (Traumatic Brain Injury Screening Questionnaire); OSU TBI-ID (Ohio State University Traumatic Brain Injury Identification Method); CPG (Chronic Pain Grade); PDHA (post-deployment health assessment); PDHRA (post-deployment health reassessment); WARCAT (Warrior Administered Retrospective Casualty Assessment Too)l; CARB (Computerized Assessment of Response Bias); PASAT (Paced Auditory Serial Addition Test); Trail AB (Trail Marking Test); WCSR4 (Wisconsin Card Sorting Test, Computerized Version 4); RAVLT (Rey Auditory Verbal Learning Test); Stroop Test;  NES 3 (Neurobehavioral Evaluation System 3); NSI (Neurobehavioral Symptom Inventory); SCAT (Sport Concussion Assessment Tool); SAC (Standardized Assessment of Concussion); BSI-18 (Brief Symptom Inventory); HVLT-R (Hopkins Verbal Learning Test-Revised) SDMT (Symbol Digit Modalities Test); COWAT (Controlled Oral Word Association Test); ACSI (Abbreviated Concussion Symptom Inventory); SUS (Slater-Usoh-Steed); SSQ (simulator sickness questionnaire); GMSVT (Green’s Medical Symptom Validity Test); VSVT (Victoria Symptom Validity Test); CVLT2 (California Verbal Learning Test-Second Edition); PCL-NM (Posttraumatic Stress Disorder Check List – non-military version); PHQ-8 (Patient Health Questionnaire); VETS (Virtual Environment TBI Screening); ASR (Acute Startle Reaction); PSQI (Pittsburgh Sleep Quality Index); DSI (Deployment Stress Inventory); SRP (Soldier Readiness Process).

*Figure S12: Categories of constructs being measured by secondary outcome measures (n=48)*


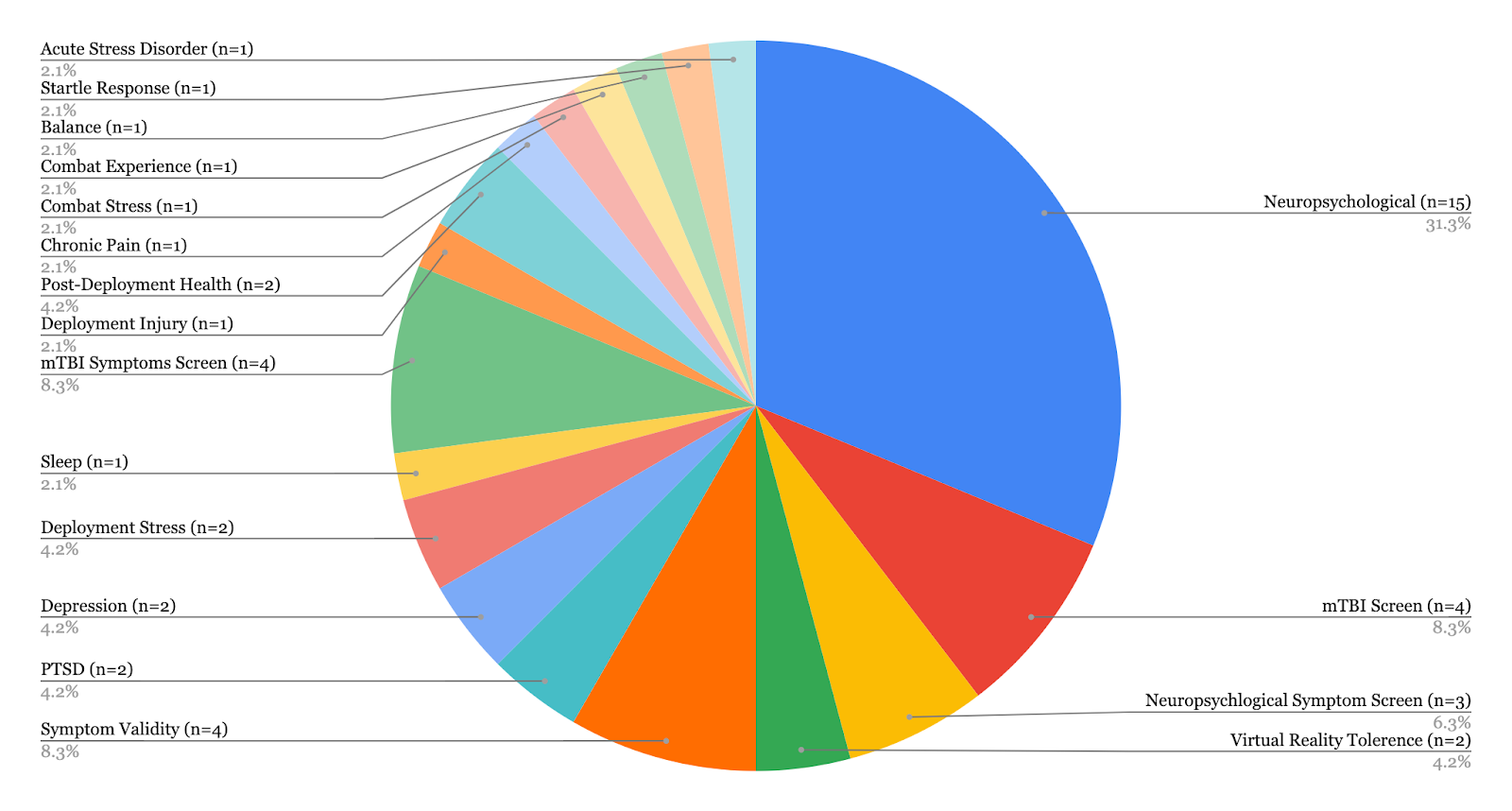


*Figure S13: Quantitative study types by papers (n=36)*


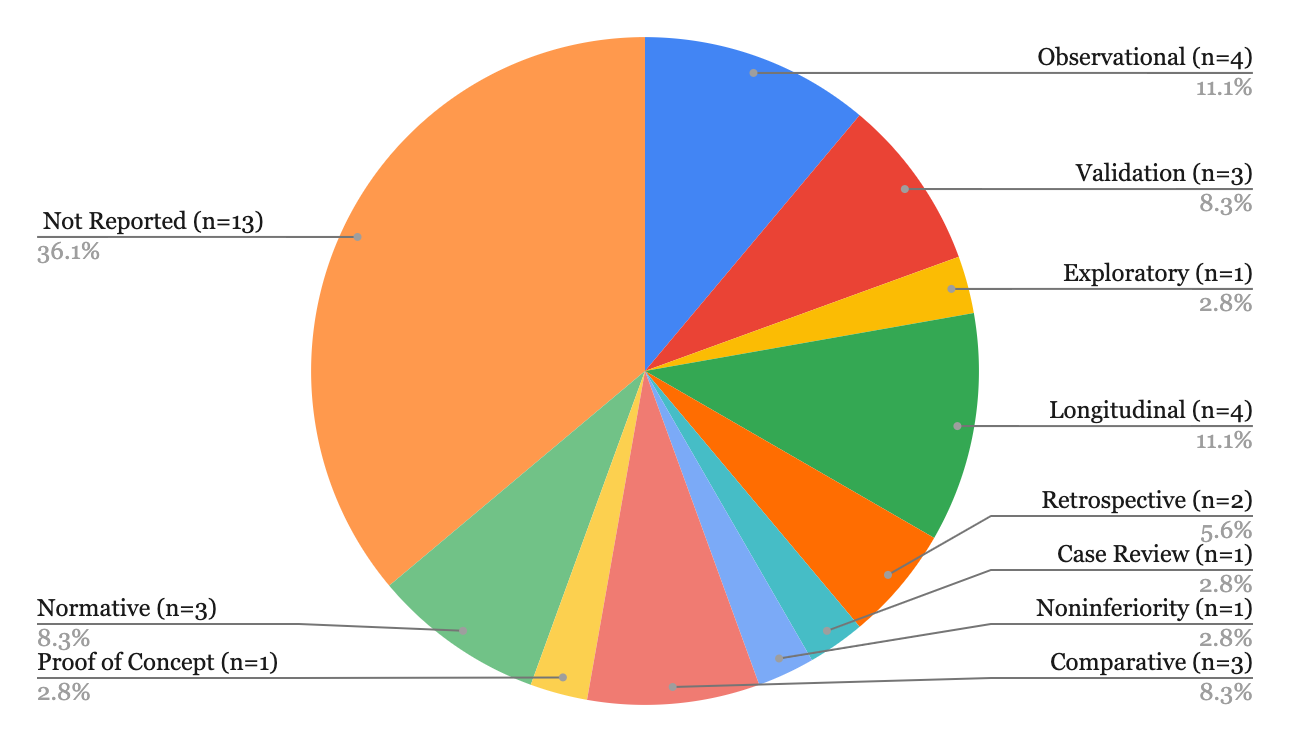


*Figure S14: Main constructs being measured by studies included in scoping review (n=41)*


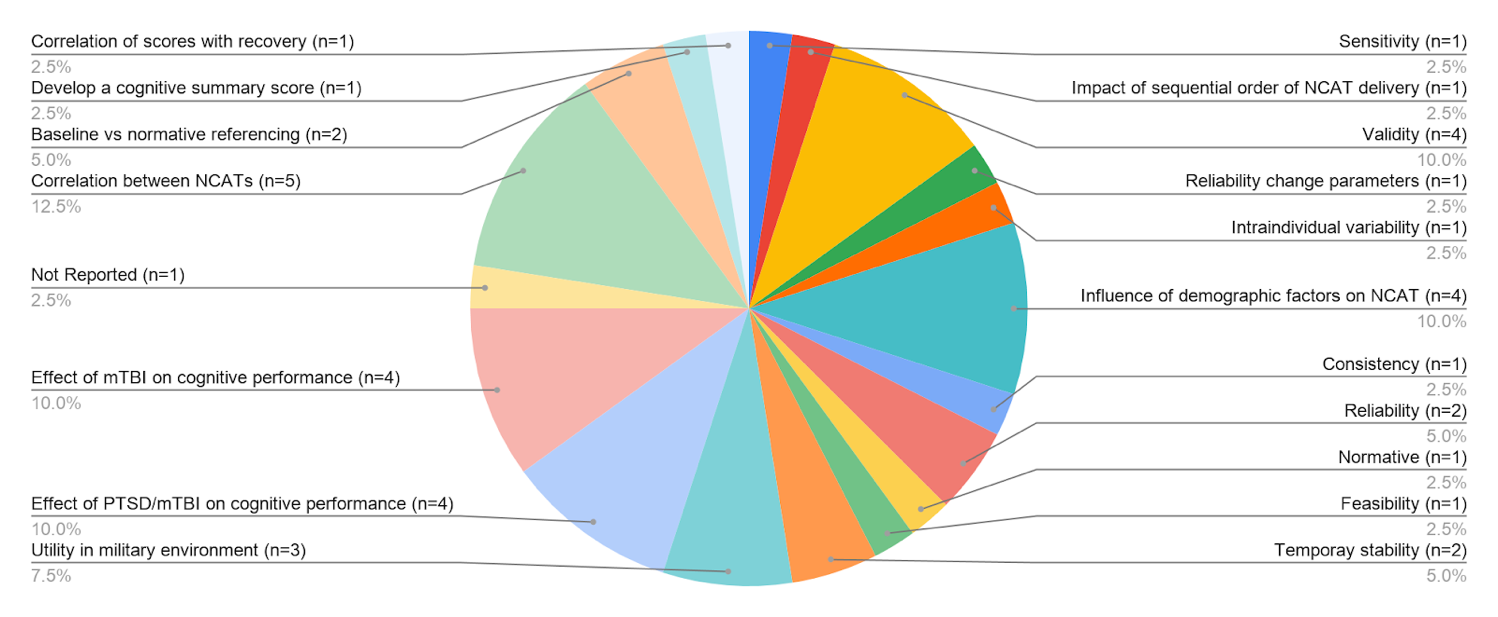


*Figure S15: Statistical analysis strategies (n=104)*


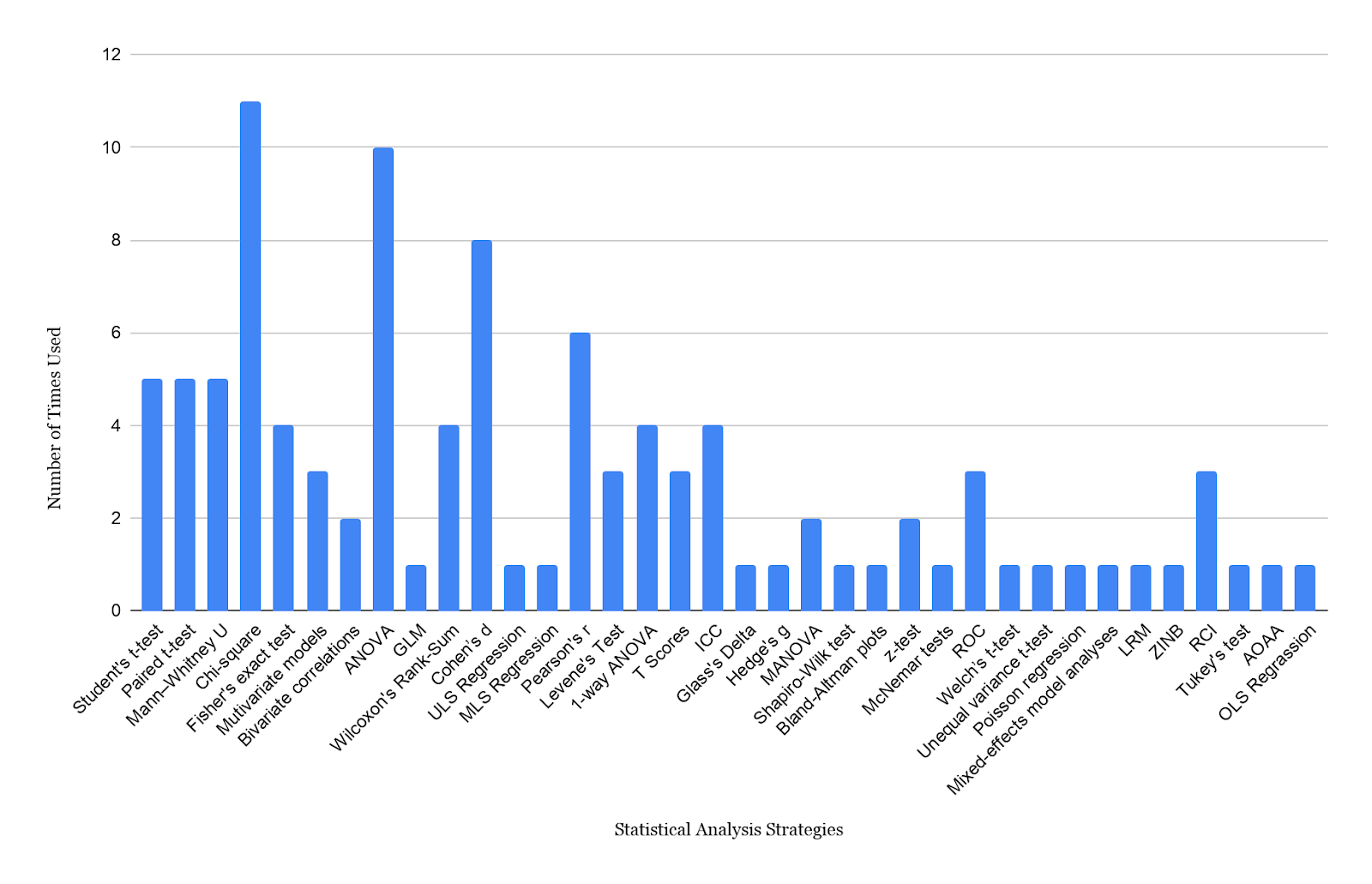


ANOVA (analysis of variance); GLM (general linear model); ULS Regression (univariate leads square regression); MLS Regression (multivariate least square regression); ICC (intraclass correlation coefficients); MANOVA (multivariate analysis of variance); ROC (receiver operator characteristic); LRM (linear regression model); RCI (reliable change index); AAOA (all others as anchors); OLS Regression (ordinary least squares regression).
